# Greenspace Exposure and the Incidence of Ocular and Adnexal Diseases: Novel Findings from a Large Prospective Cohort Study

**DOI:** 10.34133/hds.0428

**Published:** 2026-03-03

**Authors:** Zhuo-Hao Wang, Xiao-Qi Zhu, Elaine Fuertes, Hui-Yun Chen, Hui-Ling Qiu, Gang-Long Zhou, Yu-Ting Xie, Lu Wang, Jian-Cheng Jiang, Tian-Yu Zhao, Yu-Zhou Yang, Wen Chen, Joachim Heinrich, Zhou-Bin Zhang, Bo-Yi Yang

**Affiliations:** ^1^ Department of Occupational and Environmental Health, School of Public Health, Sun Yat-sen University, Guangzhou 510080, China.; ^2^ National Heart and Lung Institute, Imperial College London, London, UK.; ^3^ MRC Centre for Environment and Health, Imperial College London, London, UK.; ^4^ School of Public Health, Guangzhou Medical University and Guangzhou Center for Disease Control and Prevention, Guangzhou, China.; ^5^ Institute and Clinic for Occupational, Social and Environmental Medicine, LMU University Hospital Munich, Comprehensive Pneumology Centre (CPC) Munich, German Centre for Lung Research (DZL), Munich, Germany.; ^6^ Allergy and Lung Health Unit, Melbourne School of Population and Global Health, The University of Melbourne, Melbourne, Victoria, Australia.; ^7^ Guangzhou Joint Research Center for Disease Surveillance and Risk Assessment, Guangzhou Center for Disease Control and Prevention, Guangzhou 510440, China.; ^8^ Institute of Public Health, Guangzhou Medical University & Guangzhou Center for Disease Control and Prevention, Guangzhou 511436, China.; ^9^ Guangdong Provincial Engineering Technology Research Center of Environmental and Health Risk Assessment, Department of Occupational and Environmental Health, School of Public Health, Sun Yat-sen University, Guangzhou 510080, China.

## Abstract

**Background:** Evidence concerning greenspace and ocular and adnexal diseases (OADs) is scarce. **Methods:** This prospective cohort study followed 241,576 UK participants from baseline (2006–2010, age range: 37 to 73 years old) until May to October 2022, identifying 9 OAD subtypes through electronic health records. Residential greenspace was evaluated using the percentage of greenspace (GS%) derived from the 2005 General Land Use Database. Cox proportional hazards models and mediation analyses were employed to assess the associations. **Results:** The associations between greenspace and OAD varied by subtype, exhibiting nearly inverse J-shaped or inverse U-shaped exposure–response curves. Comparing the highest with the lowest quartile of GS% within a 300-m buffer, beneficial associations were observed for (a) lens disorders [hazard ratio (HR): 0.931 [95% confidence interval (CI): 0.896 to 0.968]] and (b) choroid and retina disorders (HR: 0.935 [95% CI: 0.874 to 1.000]). These beneficial associations for lens disorders were partially mediated by physical activity and air pollution. In contrast, detrimental associations were found for (a) eyelid, lacrimal system, and orbit disorders (HR: 1.082 [95% CI: 1.018 to 1.150]) and (b) conjunctiva disorders (first quartile versus third quartile, HR: 1.129 [95% CI: 1.027 to 1.241]). **Conclusions:** Greenspace exposure may exert both beneficial and detrimental associations across various OAD subtypes. The findings may inform the development of area- or individual-level greenspace interventions to mitigate OAD burden.

## Introduction

Ocular and adnexal diseases (OADs) are the primary etiologies of blindness and vision loss, serving as risk factors for not only various chronic diseases but also reduced quality of life [1,2]. According to the Global Burden of Disease (GBD) in 2019, blindness and vision loss accounted for 22.6 million global years lived with disability (YLDs) [3]. Besides, it is projected that by 2050, approximately 474 million people will face moderate to severe visual impairment and 61 million will be blind [4]. Nevertheless, it merits attention that around half of the global cases of visual impairment were preventable or have yet to receive appropriate intervention [5]. Hence, formulating prevention and intervention strategies targeting OAD can help to mitigate the burden of vision impairment.

The etiology of most OAD is complicated. Genetic background, unhealthy behaviors, and other diseases like hypertension and diabetes are well-documented risk factors for OAD [6–8]. In recent years, the role of outdoor environmental factors has attracted substantial attention. Greenspace, consisting of grassy meadows, forests, parks, and road green belts, is a critical part of the human environment and has been linked to various health outcomes [9]. Mechanistically, exposure to greenspace is associated with OAD by lowering air pollution levels and encouraging physical activity [9,10], both of which are protective factors for several OAD subtypes, including myopia, cataracts, and diabetic retinopathy [11–14]. Additionally, recent evidence indicates that greenspace exposure can boost beneficial microbial diversity [15], which potentially promotes ocular health by influencing the gut microbiota [16]. However, greenspace may also increase exposure to pollen-derived allergens and to ultraviolet radiation, which can lead to ocular complications and increase the risk of conjunctiva disorders, cataracts, and macular degeneration [17–19]. Therefore, it is biologically plausible to hypothesize that greenspace exposure is related to OAD development.

Although a small number of epidemiological studies have estimated the potential associations of greenspace exposure on OAD, only a few subtypes of OAD were considered (i.e., myopia, astigmatism, and diabetic retinopathy) and these studies were geographically limited (i.e., China and Spain) [20–25]. Moreover, most studies adopted a cross-sectional design with low causal inference. Considering the present evidence, it remains uncertain whether and how greenspace exposure can be utilized as an intervention measure for OAD.

Therefore, we here comprehensively estimated associations between greenspace exposure and the incidence of OAD subtypes using a large prospective cohort of UK adults. Further, we explored potential mechanisms by which greenspace could be associated with OAD incidence. Findings from our study would be useful for developing population- or individual-level intervention strategies for mitigating the burden of OAD.

## Materials and Methods

### Study population and design

Between March 2006 and October 2010, the UK Biobank project recruited over 500,000 baseline participants aged 37 to 73 years old from 22 assessment centers across England, Wales, and Scotland [26]. During the baseline survey, a wide range of data encompassing sociodemographic characteristics, lifestyle factors, and medical history were collected using methods such as touchscreen questionnaires, verbal interviews, anthropometric measurements, and biological sampling. For the current analysis, the follow-up period commenced from the date of the baseline assessment. In England, participants were followed up until 2022 October 31; in Wales, the follow-up lasted until 2022 May 31; and in Scotland, it extended until 2022 August 31 [27]. The specific variables from the UK Biobank used in this study were summarized in Table S1. The UK Biobank obtained ethical approval from the North West Multi-center Research Ethics Committee (MREC) (REC reference: 21/NW/0157). This research has been conducted with the UK Biobank Resource under project 90798. This study is reported in accordance with the Strengthening the Reporting of Observational Studies in Epidemiology (STROBE) guideline (Checklist S1).

### OAD definition and identification

OADs were defined as H00-H59 according to the International Classification of Diseases, 10th Revision (ICD-10), including 11 subtypes of OAD (Table S2) [28]. We determined OAD incident and its corresponding date by referencing the date of “first occurrence” recorded in electronic health records. These records were derived from multiple sources, including primary care data, hospital inpatient data, death registration records, baseline records, and self-reported medical conditions during follow-ups (Table S3) [29].

We identified baseline patients based on 2 criteria: (a) Individuals whose “first occurrence” date of OAD was before the date of their attendance at the assessment center were defined as baseline patients. (b) During the completion of the baseline electronic touchscreen questionnaire, individuals who selected “left eye”, “right eye”, or “both eyes” in any of the following questions: “Which eye(s) are affected by myopia (short-sightedness), hypermetropia (long-sightedness), presbyopia, astigmatism, strabismus (squint), amblyopia (lazy eye), diabetes-related eye disease, glaucoma, injury or trauma resulting in vision loss, cataract, macular degeneration, or other eye conditions?” were also classified as baseline patients. All baseline patients were excluded from our analysis.

### Greenspace assessment

The percentage of greenspace (GS%) surrounding participants’ residences was our exposure. Specifically, the GS% was calculated using data from the 2005 General Land Use Database (GLUD) at the 2001 Census Output Area level [30], which divided the land into 9 types including greenspace, domestic and nondomestic buildings, gardens, roads, paths, rail, water bodies, and other land uses. The residential GS% was defined as the proportion of greenspace to the total land area within a specific buffer zone around residential addresses. Based on evidence of greenspace–health links and greenspace accessibility policies [31–33], we used 300- and 1,000-m buffers to represent nearby and wider-area greenspaces relative to participants’ homes, respectively.

### Confounders and mediators

We considered variables that met the following criteria as potential confounding factors: (a) the variable must be a factor influencing ocular health; (b) the variable must be associated with greenspace; (c) the variable must not be an intermediate factor between greenspace and OAD. To effectively identify confounders and explore potential mediators, directed acyclic graphs (DAGs; DAGitty v3.1 software, website: www.dagitty.net) were employed (Fig. S1) [34]. According to the DAGs, we selected the following minimal set of confounding variables: age (in years), sex (male or female), ethnicity (white or non-white), Thompson deprivation index (TDI; low, mild, moderate, highly, and severely deprivation), qualifications (None; O levels, GCSEs, or CSEs; A levels or AS levels; NVQ, HND, HNC, or other professional qualification; college or university degree), employment status (employed; retired; unemployed, home maker, or other), and household income (<£18,000; £18,000 to £30,999; £31,000 to £51,999; ≥£52,000) [35]. These variables were obtained by questionnaire during the initial visit to the assessment center, except for the TDI, a composite index of household overcrowding, unemployment, non-homeownership, and non-car ownership [36], which was obtained from the local primary care trust registry, and it is used to evaluate the socioeconomic deprivation at the community or regional level.

Furthermore, based on the DAGs, participants’ physical activity levels [assessed by weekly metabolic equivalent of task (MET) minutes] and air pollution within their residential areas [i.e., inhalable particulate matter with aerodynamic diameter less than 2.5 μm (PM_2.5_), inhalable particulate matter with aerodynamic diameter less than 10 μm (PM_10_), and nitrogen dioxide (NO_2_), all assessed using a land use regression (LUR) model] were selected as potential mediators [37].

### Statistical analyses

Continuous variables that meet the Anderson–Darling normality test were presented as means with standard deviations (SDs); otherwise, medians and interquartile ranges (IQRs) were used. Frequencies with percentages were used for categorical variables. Spearman correlation analysis was used to assess correlations between greenspace and various mediators (Table S4).

We employed Cox proportional hazards models to examine associations between GS% within 300-m buffer and the incidence of the OAD subtypes. Subtypes with a cumulative incidence below 1% were excluded (to mitigate the issue of low statistical power). Prior to formal analysis, the proportional hazards assumption was tested (for details, refer to Tables S5 and S6). For GS%, if the *P* value for the Schoenfeld residual method was less than 0.05, a time interaction term (x*t) was added. For covariates that violated the assumption, stratification was applied to these covariates within the model (model details are shown in Tables S7 to S9) [38]. Association estimates [hazard ratio (HR) and 95% confidence interval (CI)] are presented by quartiles, given the nonlinearity in almost all exposure–outcome curves (tested using restricted cubic spline; *P*-overall < 0.05 for overall significance; *P*-nonlinear < 0.05 for nonlinear significance) [39]. Additionally, we fitted models using greenspace within a 1,000-m buffer to estimate the association of wider-area greenspace. We fitted both crude and main models adjusted by confounders selected using DAG.

For those OAD subtypes with significant associations, we estimated the population attributable fraction (PAF) of green space according to the following formula: PAF = [(*HR* − 1)/*HR*] * *Pe*, where *Pe* represents the proportion of the population exposed to the exposed factor.

We conducted a series of sensitivity analyses to ensure the robustness of the results. Specifically, we first fitted a mixed-effect Cox model with the assessment center as a random term. Second, we further included body mass index (<25 kg/m^2^; ≥25, <30 kg/m^2^; ≥30 kg/m^2^), current smoking status (yes or no), current alcohol consumption (yes or no), history of hypertension (yes or no), and history of diabetes (yes or no) to evaluate the impact of these common individual factors, which were excluded by the DAG. Third, we removed the participants who had more than one subtype of OAD to mitigate the interactions within different subtypes of OAD. Fourth, we excluded the participants who experienced OAD events within 2 years of follow-up to mitigate reverse causation. Firth, we defined death as a competing event and constructed a Fine-Gray model and cause-specific hazards model (using the “riskRegression” R package) [40]. Sixth, we utilized the Multiple Imputation by Chained Equations handling missing data to validate the robustness of our findings (using the “MICE” R package).

Finally, we employed VanderWeele’s decomposition causal mediation analyses (using the “CMAverse” R package; details in Supplementary Methods) to estimate if any observed beneficial associations between greenspace and specific OAD subtypes can be explained by increased physical activity and lower air pollution levels [41]. We decomposed the total effect (TE) of green space on specific OAD subtypes into the total natural direct effect (DE) and total natural indirect effect (NIE). The proportion mediated was subsequently calculated as the percentage of the NIE relative to the TE. A resampling method (bootstrap) was used to estimate the CIs and *P* values for these effects.

All statistical analyses were performed using R software version 4.3.0, and a *P* value less than 0.05 in a 2-tailed test indicates statistical significance.

## Results

### Characteristics of the study participants

**Fig. 1.**
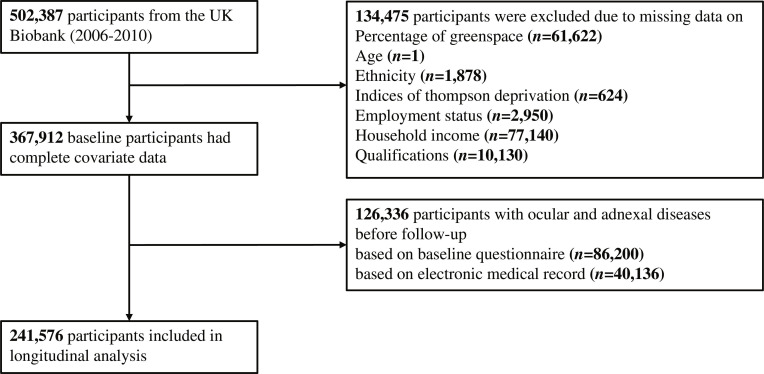
Flow chart of study participant selection.

Initially, 502,387 UK Biobank participants were enrolled. After excluding 134,475 individuals with missing data on GS%, main covariates, and 126,336 baseline OAD cases, we finally included 241,576 participants into the current analysis (Fig. 1). The basic characteristics of the excluded participants were generally comparable to those included in the analysis (Table S10).

The median (IQR) age of the included participants was 56.0 (13.0) years old. Among them, 51.7% were female, and 94.4% identified as white (Table 1). During 12 to 15 years of follow-up, a total of 43,114 participants (17.85%) newly developed OAD. The 3 most common OAD subtypes were disorders of the lens (22,787 cases, 9.43%), disorders of the eyelid, lacrimal system and orbit (9,293 cases, 3.84%), and disorders of the choroid and retina (8,160 cases, 3.38%) (Table S2). OAD cases were more likely to be older, female, and from higher deprivation backgrounds than those who did not develop any OAD. Greenspace exposure levels varied substantially across participants. The median GS% (IQR) within 300- and 1,000-m buffers was 31.3 (32.6) and 44.0 (33.6), respectively, among all participants (Table 1).

**Table 1.** Characteristics of the study participants

Characteristic	Participants, no. (%)			*P* value
	All (*n* = 241,576)	Without OAD (*n* = 198,462)	With OAD (*n* = 43,114)	
Demographic characteristics				
Age				
Median (IQR), years	56.0 (13.0)	55.0 (13.0)	61.0 (10.0)	<0.001
Sex				
Male	116,675 (48.3)	96,978 (48.9)	19,697 (45.7)	<0.001
Female	124,901 (51.7)	101,484 (51.1)	23,417 (54.3)	
Ethnic				
White	228,119 (94.4)	187,419 (94.4)	40,700 (94.4)	0.784
Non-white	13,457 (5.6)	11,043 (5.6)	2,414 (5.6)	
Body mass index				
<25 kg/m^2^	81,140 (33.6)	68,146 (34.3)	12,994 (30.1)	<0.001
≥25, <30 kg/m^2^	102,924 (42.6)	84,390 (42.5)	18,534 (43.0)	
≥30 kg/m^2^	56,621 (23.4)	45,237 (22.8)	11,384 (26.4)	
Missing	891 (0.4)	689 (0.3)	202 (0.5)	
Socioeconomic characteristics				
Thompson deprivation index				
Low deprivation	48,416 (20.0)	39,870 (20.1)	8,546 (19.8)	0.014
Mildly deprivation	48,215 (20.0)	39,636 (20.0)	8,579 (19.9)	
Moderately deprivation	48,324 (20.0)	39,686 (20.0)	8,638 (20.0)	
Highly deprivation	48,309 (20.0)	39,825 (20.1)	8,484 (19.7)	
Severely deprivation	48,312 (20.0)	39,445 (19.9)	8,867 (20.6)	
Qualifications				
None	35,751 (14.8)	26,881 (13.5)	8,870 (20.6)	<0.001
O levels, GCSEs, or CSEs	132,798 (55.0)	111,940 (56.4)	20,858 (48.4)	
A levels or AS levels	10,074 (4.2)	8,307 (4.2)	1,767 (4.1)	
NVQ, HND, HNC, or other professional qualification	29,030 (12.0)	23,111 (11.6)	5,919 (13.7)	
College or university degree	33,923 (14.0)	28,223 (14.2)	5,700 (13.2)	
Employment status				
Employed	154,882 (64.1)	134,279 (67.7)	20,603 (47.8)	<0.001
Retired	68,266 (28.3)	48,870 (24.6)	19,396 (45.0)	
Unemployed, home maker, or other	18,428 (7.6)	15,313 (7.7)	3,115 (7.2)	
Household income				
<£18,000	53,479 (22.1)	40,321 (20.3)	13,158 (30.5)	<0.001
£18,000–£30,999	60,681 (25.1)	48,190 (24.3)	12,491 (29.0)	
£31,000–£51,999	63,977 (26.5)	54,111 (27.3)	9,866 (22.9)	
≥£52,000	63,439 (26.3)	55,840 (28.1)	7,599 (17.6)	
Lifestyle and medical history				
The MET minutes per week for all activities				
Median (IQR), minutes	1,770 (2,746.0)	1,770 (2,736.0)	1,750 (2,796.0)	0.045
Missing	39,204 (16.2)	31,568 (15.9)	7,636 (17.7)	
Diabetes history				
No	231,431 (95.8)	191,792 (96.6)	39,639 (91.9)	<0.001
Yes	10,145 (4.2)	6,670 (3.4)	3,475 (8.1)	
Hypertension history				
No	182,393 (75.5)	153,452 (77.3)	28,941 (67.1)	<0.001
Yes	59,183 (24.5)	45,010 (22.7)	14,173 (32.9)	
Current smoking				
No	215,300 (89.1)	176,646 (89.0)	38,654 (89.7)	<0.001
Yes	26,267 (10.9)	21,809 (11.0)	4,458 (10.3)	
Missing	9 (0.0)	7 (0.0)	2 (0.0)	
Current drinking				
No	16,782 (6.9)	13,088 (6.6)	3,694 (8.6)	<0.001
Yes	224,783 (93.0)	185,365 (93.4)	39,418 (91.4)	
Missing	11 (0.0)	9 (0.0)	2 (0.0)	
Environmental factors				
Greenspace percentage within 300-m buffer				
Median (IQR), %	31.3 (32.6)	31.1 (32.7)	32.1 (32.0)	<0.001
Greenspace percentage within 1,000-m buffer				
Median (IQR), %	44.0 (33.6)	43.8 (33.8)	44.8 (32.7)	<0.001
PM_2.5_				
Median (IQR), μg/m^3^	10.0 (1.4)	10.0 (1.4)	10.0 (1.3)	0.013
Missing	2,819 (1.2)	2,424 (1.2)	395 (0.9)	
PM_10_				
Median (IQR), μg/m^3^	16.0 (1.8)	16.0 (1.8)	15.9 (1.8)	0.001
Missing	2,819 (1.2)	2,424 (1.2)	395 (0.9)	
NO_2_				
Median (IQR), μg/m^3^	25.6 (10.0)	25.6 (10.1)	25.6 (9.6)	0.584
Missing	2,660 (1.1)	2,275 (1.1)	385 (0.9)	

OAD, ocular and adnexal diseases; IQR, interquartile range; O levels, Ordinary levels; GCSE, General Certificate of Secondary Education; CSE, Certificate of Secondary Education; A levels, Advanced levels; AS levels, Advanced Subsidiary levels; NVQ, National Vocational Qualification; HND, Higher National Diploma; HNC, Higher National Certificate; MET minutes, metabolic equivalent of task minutes are obtained by multiplying the MET value of physical activity by its duration; PM_2.5_, particles with an aerodynamic diameter of ≤ 2.5 μm; PM_10_, particles with an aerodynamic diameter of ≤ 10 μm; NO_2_, nitrogen dioxide

### Associations between greenspace and OAD

**Fig. 2.**
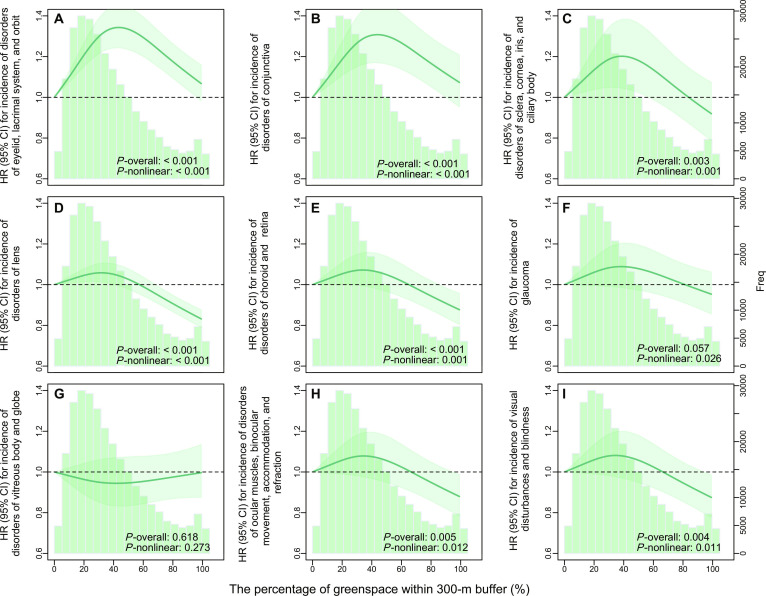
The exposure–response curves between the percentage of greenspace within 300-m buffer and the incidence of 9 ocular and adnexal disease subtypes. Note: The models were adjusted for age, gender, ethnicity, indices of Thompson deprivation, employment status, household income, and qualifications. HR, hazard ratio; CI, confidence interval. The *P*-overall value indicated the significance level of the overall association of all factors in the model (significant if less than 0.05). The *P*-nonlinear value indicated the significance level of the nonlinear association in the model (significant if less than 0.05). (A) Disorders of eyelid, lacrimal system, and orbit. (B) Disorders of conjunctiva. (C) Disorders of sclera, cornea, iris, and ciliary body. (D) Disorders of lens. (E) Disorders of choroid and retina. (F) Glaucoma. (G) Disorders of vitreous body and globe. (H) Disorders of ocular muscles, binocular movement, accommodation, and refraction. (I) Visual disturbances and blindness.

The associations between greenspace and OAD differed by its subtype (Table 2), with nearly all exposure–response relationships exhibiting an inverse J-shaped/inverse U-shaped curve (Fig. 2). Specifically, we observed that higher levels of greenspace were associated with a reduced risk of 2 OAD subtypes. The adjusted HRs for (a) disorders of the lens and (b) disorders of choroid and retina, comparing the highest to the lowest quartiles of GS% within 300-m buffer, were 0.931 (95% CI: 0.896 to 0.968) and 0.935 (95% CI: 0.874 to 1.000), respectively. However, we also observed that higher levels of greenspace were associated with increased risk of 2 OAD subtypes. Specifically, comparing participants in the highest to the lowest quartiles of GS% within 300-m buffer, the adjusted HR for disorders of the eyelid, lacrimal system, and orbit was 1.082 (95% CI: 1.018 to 1.150). Additionally, when comparing participants in the third quartile to those in the first quartile, the adjusted HR for disorders of the conjunctiva was 1.129 (95% CI: 1.027 to 1.241). Notably, for disorders of ocular muscles, binocular movement, accommodation, and refraction, the association with greenspace was borderline marked within 1,000-m buffer when comparing the highest to the lowest quartiles of GS% (HR: 0.827 [0.666, 1.028]). However, no significant associations were found for (a) glaucoma; (b) disorders of the vitreous body and globe; (c) disorders of the sclera, cornea, iris, and ciliary body; and (d) visual disturbances and blindness.

**Table 2.** The association between the percentage of greenspace within 300- and 1,000-m buffers and the incidence of ocular and adnexal diseases. *P < 0.05.

OAD subtypes		GS% within 300-m buffer		GS% within 1,000-m buffer	
		HR (95% CI)	aHR ^a^ (95% CI)	HR (95% CI)	aHR (95% CI)
Disorders of lens (*n* = 22,787)	Q1 ^b^	Ref.			
	Q2	1.031 (0.994, 1.070)	0.985 (0.949, 1.023)	1.070 (1.032, 1.111)*	0.990 (0.954, 1.028)
	Q3	1.066 (1.028, 1.106)*	0.999 (0.963, 1.037)	1.101 (1.062, 1.142)*	1.004 (0.966, 1.043)
	Q4	0.991 (0.955, 1.029)	0.931 (0.896, 0.968)*	1.012 (0.975, 1.051)	0.917 (0.881, 0.954)*
Disorders of choroid and retina (*n* = 8,160)	Q1	Ref.			
	Q2	1.041 (0.979, 1.107)	0.997 (0.935, 1.063)	1.048 (0.985, 1.114)	0.988 (0.926, 1.054)
	Q3	1.077 (1.013, 1.145)*	1.010 (0.947, 1.077)	1.062 (0.999, 1.129)	0.985 (0.922, 1.053)
	Q4	1.001 (0.940, 1.065)	0.935 (0.874, 1.000)*	1.001 (0.940, 1.065)	0.921 (0.859, 0.987)*
Disorders of eyelid, lacrimal system, and orbit (*n* = 9,293)	Q1	Ref.			
	Q2	1.071 (1.010, 1.137)*	1.039 (0.979, 1.103)	1.143 (1.077, 1.213)*	1.105 (1.040, 1.174)*
	Q3	1.208 (1.141, 1.280)*	1.164 (1.098, 1.234)*	1.263 (1.192, 1.339)*	1.226 (1.153, 1.303)*
	Q4	1.108 (1.045, 1.175)*	1.082 (1.018, 1.150)*	1.163 (1.096, 1.234)*	1.139 (1.069, 1.214)*
Disorders of conjunctiva (*n* = 3,936)	Q1	Ref.			
	Q2	1.032 (0.942, 1.130)	1.005 (0.917, 1.102)	1.239 (1.132, 1.356)*	1.208 (1.100, 1.326)*
	Q3	1.162 (1.064, 1.270)*	1.129 (1.027, 1.241)*	1.155 (1.053, 1.265)*	1.127 (1.018, 1.247)*
	Q4	1.095 (1.002, 1.198)*	1.079 (0.943, 1.234)	1.225 (1.119, 1.341)*	1.198 (1.053, 1.363)*
Disorders of ocular muscles, binocular movement, accommodation, and refraction (*n* = 4,757)	Q1	Ref.			
	Q2	0.993 (0.916, 1.077)	0.963 (0.887, 1.044)	0.981 (0.906, 1.062)	0.936 (0.862, 1.016)
	Q3	1.060 (0.979, 1.148)	1.028 (0.948, 1.114)	0.972 (0.897, 1.052)	0.918 (0.834, 1.010)
	Q4	0.969 (0.893, 1.051)	0.955 (0.877, 1.039)	0.898 (0.828, 0.973)*	0.827 (0.666, 1.028)
Glaucoma (*n* = 5,165)	Q1	Ref.			
	Q2	1.032 (0.942, 1.130)	1.010 (0.932, 1.093)	1.072 (0.991, 1.160)	1.028 (0.948, 1.114)
	Q3	1.162 (1.064, 1.270)*	1.032 (0.943, 1.129)	1.107 (1.024, 1.196)*	1.031 (0.939, 1.133)
	Q4	1.095 (1.002, 1.198)	0.885 (0.715, 1.096)	1.096 (1.014, 1.185)*	0.934 (0.751, 1.161)
Disorders of vitreous body and globe (*n* = 3,397)	Q1	Ref.			
	Q2	0.879 (0.797, 0.969)*	0.870 (0.788, 0.961)*	0.996 (0.905, 1.097)	0.955 (0.865, 1.055)
	Q3	1.034 (0.941, 1.135)	1.018 (0.919, 1.127)	1.007 (0.915, 1.108)	0.943 (0.847, 1.051)
	Q4	1.028 (0.936, 1.129)	1.029 (0.882, 1.200)	1.074 (0.977, 1.180)	0.982 (0.848, 1.138)
Disorders of sclera, cornea, iris, and ciliary body (*n* = 2,643)	Q1	Ref.			
	Q2	1.065 (0.954, 1.188)	1.018 (0.910, 1.139)	1.188 (1.067, 1.324)*	1.128 (1.009, 1.262)*
	Q3	1.133 (1.018, 1.262)*	1.080 (0.966, 1.208)	1.121 (1.005, 1.250)*	1.046 (0.931, 1.175)
	Q4	1.037 (0.929, 1.158)	1.015 (0.904, 1.141)	1.022 (0.914, 1.143)	0.981 (0.869, 1.109)
Visual disturbances and blindness (*n* = 4,512)	Q1	Ref.			
	Q2	1.052 (0.969, 1.142)	1.018 (0.937, 1.106)	1.013 (0.934, 1.098)	1.000 (0.920, 1.086)
	Q3	1.056 (0.973, 1.146)	1.045 (0.961, 1.136)	0.951 (0.876, 1.033)	0.980 (0.899, 1.069)
	Q4	0.929 (0.854, 1.011)	0.988 (0.905, 1.080)	0.914 (0.841, 0.993)*	0.989 (0.904, 1.082)

OAD, ocular and adnexal diseases; GS%, percentage of greenspace; HR, hazard ratio; CI, confidence interval; Ref, reference

^a^ Adjusted model includes age, gender, ethnicity, indices of Thompson deprivation, employment status, household income, and qualifications.

^b^ The ranges for Q1 to Q4 for GS% in the 300-m buffer were “0 to 18.4, 18.4 to 31.3, 31.3 to 51.0, 51.0 to 99.2”, and for the 1,000-m buffer, they were “0 to 29.0, 29.0 to 44.0, 44.0 to 62.6, 62.6 to 99.2”, respectively.

The observed associations remained largely consistent in several sensitivity analyses: (a) alternatively fitted GS% within a larger buffer (1,000 m) (Table 2); (b) adjusted for additional potential covariates (Table S11); (c) excluded incidents within the first 2 years of follow-up (Table S12); (d) excluded patients with multiple OADs (Table S13); (e) accounted for competing risks (Table S14); and (f) handled the missing data (Table S15). However, after adjusting for the center effects, the conditional associations of GS% within 1,000-m buffer (i.e., the highest quartile versus the lowest quartile within the same center) with disorders of ocular muscles, binocular movement, accommodation and refraction (HR: 0.665 [0.528, 0.839]), glaucoma (HR: 1.355 [1.138, 1.613]), and disorders of the vitreous body and globe (HR: 1.458 [1.198, 1.773]) became significant (Table S16).

### PAF of greenspace for specific OAD subtypes

The PAF results indicated that for lens disorders, comparing participants in the highest to the lowest quartiles of GS% within 300-m buffer, the PAF was 1.72% (95% CI: 0.85%, 2.76%; *P* < 0.001). Regarding choroid and retina disorders, we found that the PAF was 1.98% (95%CI: 0.47%, 3.91%; *P* = 0.020) when comparing the highest to the lowest quartiles of GS% within 1,000-m buffer. Conversely, for disorders of the eyelid, lacrimal system, and orbit, as well as disorders of the conjunctiva, when comparing participants in the higher quartiles of GS% within 300-m buffer, the corresponding PAFs were 1.90% (95% CI: 0.56%, 3.65%; *P* = 0.024 [highest quartile versus lowest quartile]) and 2.85% (0.75%, 5.62%; *P* = 0.014 [third quartile versus first quartile]), respectively. Similar results were also found in the 1,000-m buffer zone (Table S17).

### Potential mediating effects of greenspace–OAD

In mediation analyses, we decomposed the TE into natural DE and NIE with the comparison between the highest (Q4) to the lowest (Q1) GS% quartile. For disorders of the lens, physical activity mediated 3.5% of the association with GS%, while air pollutants (PM_2.5_, PM_10_, NO_2_) mediated a substantial proportion, ranging from 22.5% to 152.2% (Table 3). No significant mediation was observed for disorders of the choroid and retina.

**Table 3.** The effect decomposition of different mediators under the percentage of green space exposure within 300-m buffer. Effect estimates were calculated by the “CMAverse” package in R software based on mediating factors for each 1-unit increase. Proportion mediated = [exp(natural indirect effect)/exp(natural indirect effect * direct effect)]*100%. *P < 0.05.

OAD subtypes	Mediators		Total effect (HR [95% CI])	Decomposition of total effect ^a^		Proportion mediated, %
				Direct effect (HR [95% CI])	Natural indirect effect (HR [95% CI])	
Disorders of lens	MET mins (*n* = 202,372)	Q1 vs. Q4	0.926 (0.888, 0.964)*	0.929 (0.891, 0.967)*	0.997 (0.995, 0.999)*	3.5 (1.3, 9.9)*
	PM_2.5_ (*n* = 238,757)	Q1 vs. Q4	0.930 (0.895, 0.971)*	0.937 (0.888, 0.986)*	0.898 (0.851, 0.949)*	152.2 (59.2, 402.4)*
	PM_10_ (*n* = 238,757)	Q1 vs. Q4	0.924 (0.884, 0.959)*	0.905 (0.860, 0.952)*	0.979 (0.965, 0.998)*	25.5 (2.2, 68.4)*
	NO_2_ (*n* = 238,916)	Q1 vs. Q4	0.930 (0.885, 0.971)*	0.964 (0.905, 1.025)	0.903 (0.854, 0.943)*	143.8 (63.7, 384.3)*
Disorders of choroid and retina	MET mins (*n* = 202,372)	Q1 vs. Q4	0.952 (0.895, 1.034)	0.959 (0.901, 1.042)	0.993 (0.990, 0.996)*	13.3 (−71.3, 127.2)
	PM_2.5_ (*n* = 238,757)	Q1 vs. Q4	0.962 (0.897, 1.024)	0.915 (0.824, 1.011)	0.909 (0.844, 1.001)	253.9 (−1,845.2, 1,984.4)
	PM_10_ (*n* = 238,757)	Q1 vs. Q4	0.958 (0.894, 1.029)	0.953 (0.886, 1.036)	0.969 (0.939, 1.001)	74.3 (−506.0, 636.6)
	NO_2_ (*n* = 238,916)	Q1 vs. Q4	0.966 (0.900, 1.027)	0.942 (0.845, 1.021)	0.916 (0.855, 1.001)	257.4 (−1,610.7, 1,902.9)

OAD, ocular and adnexal diseases; HR, hazard ratio; CI, confidence interval; MET minutes, metabolic equivalent of task minutes are obtained by multiplying the MET value of physical activity by its duration; Q1 vs. Q4, the fourth quartile relative to the first quartile; PM_2.5_, particulate matter <2.5 μm in aerodynamic diameter; PM_10_, particulate matter <10 μm in aerodynamic diameter; NO_2_, nitrogen dioxide

^a^ Adjusted confounders include age, gender, ethnicity, indices of Thompson deprivation, employment status, household income, qualifications, and assessment center.

## Discussion

This work represents the first large-scale prospective cohort study to comprehensively investigate associations between greenspace exposure and the onset of 9 subtypes of OAD, as well as the mechanisms underlying these associations. Our findings reveal that greenspace may exert beneficial or detrimental associations across different subtypes of OAD, with nearly all exposure–response relationships exhibiting an inverse J-shaped/inverse U-shaped curve. Specifically, exposure to higher greenspace levels was protective for 2 OAD subtypes including disorders of the lens and disorders of choroid and retina. These associations were mediated by lower air pollutants levels and enhanced physical activity. However, adverse associations between higher greenspace exposure and the onset of 2 OAD subtypes were also observed. These included disorders of the eyelid, lacrimal system, and orbit and disorders of the conjunctiva. Furthermore, we also calculated the PAF of GS% for the aforementioned OAD subtypes and observed that exposure to higher levels of greenspace could potentially prevent 1.37% to 2.17% of disorders of the lens and 1.63% to 1.98% of disorders of the choroid and retina. Conversely, maintaining the lowest level of greenspace exposure could potentially prevent 1.9% to 4.60% of disorders of the eyelid, lacrimal system, and orbit, as well as 2.81% to 4.31% of disorders of the conjunctiva.

The beneficial associations between residential greenspace and specific OAD subtypes observed in our study are consistent with existing literature. A cross-sectional study of 484,380 Chinese adults reported that for every 0.1 increase in the normalized difference vegetation index (NDVI), diabetic retinopathy prevalence decreased by 10% [23]. Our study is, however, the first to report beneficial associations between greenspace and disorders of the lens; hence, this finding requires replication in other populations. Notably, our results also revealed a borderline-marked protective association between exposure to higher levels of greenspace and disorders of ocular muscles, binocular movement, accommodation, and refraction. A recent cohort study of Chinese adults showed that for every 0.1 increase in NDVI, the risks of developing myopia and hyperopia decreased by 33% and 32%, respectively, which also supports our findings [42].

The exact mechanisms underlying these beneficial associations are unclear, yet several hypotheses have been proposed. First, greenspace may benefit OAD by encouraging physical activity. Accumulating evidence has documented that people living in greener areas do more exercise [10], which is considered protective for ocular health by regulating blood flow [43], promoting ocular metabolism, and preventing the accumulation of metabolic waste in ocular vessels and the crystalline lens [44]. Second, beyond its environmental benefits (e.g., carbon neutrality) [45], greenspace may attract people outside and hence increase their exposure to sunlight, which can regulate the ciliary muscle and retard axial elongation of the eyeball, reducing the risk of refractive errors like myopia [11]. Third, there is some limited evidence to suggest that greenspace may reduce air pollution levels, and the latter has been linked to increased inflammatory responses and oxidative stress on the ocular system as well as damage to the function of retinal microvessels and the optic nerve [46]. These hypotheses are supported by our mediation analyses in which physical exercise mediated the associations between greenspace and the disorders of lens (Table 3). Notably, the estimated mediating proportion for PM_2.5_ and NO_2_ exceeded 100%, which may indicate the presence of suppression effect that diminishes the observed TE.

We are the first to report adverse associations of greenspace with disorders of the eyelid, lacrimal system, and orbit and disorders of conjunctiva; thus, the findings we detected cannot be compared with the results of other studies. A possible explanation may be that pollen released by plants harms ocular health in multiple ways. First, human eyes are directly exposed to pollen, which may increase the odds of conjunctivitis by triggering allergic reactions [47]. Second, stimulation from pollen can heighten the sensitivity of eyes and potentially increase the risk of ocular symptoms like itching and tearing. The eye discomfort caused by pollen can lead to habitual eye rubbing [48], which results in an infection of the eyes. Third, recent studies also highlight that pollen contains fungi-producing spores and toxins that could harm ocular health [49].

The exposure–response curve for the observed detrimental associations generally followed an inverse U-shaped curve. One possible explanation is that, at lower levels of greenspace exposure, the increase in greenspace is accompanied by an increase in allergenic pollen, leading to elevated adverse association estimates [47]. Conversely, at higher levels of greenspace exposure, which may produce substantial allergic pollens, people may take more protective measures against allergens, leading to a decrease in the association estimates. The exposure–response curve for the observed beneficial associations typically displayed an inverse J-shaped curve, indicating a threshold effect of greenspace.

Our findings provide additional evidence concerning the potential etiology of OADs, which would be helpful for health professionals, policy makers, and individuals to develop intervention strategies for mitigating the burden of OAD. Future research using advanced design (e.g., intervention studies) and among other populations should be carried out to validate our findings and to underpin potential mechanisms underlying green space and OAD.

One of the strengths of our study is that we used a longitudinal and prospective cohort study including participants from across the UK, thereby covering a wide range of geographic areas and population groups. This likely increased the reliability and generalizability of our findings. Additionally, we investigated the relationship between greenspace and nearly all categories of OAD subtypes, providing comprehensive evidence on the link between greenspace and ocular health.

There are several limitations to this study. First, the residential addresses of participants may have changed during the follow-up period, which could result in some exposure misclassification. Second, we cannot assess the quality of greenspace. Evidence suggests that factors such as the diversity of vegetation types and the morphology of greenspace can influence their attractiveness and accessibility [20]. Having information on the proportion of allergic plant species in the greenspaces would have been particularly essential to further investigate the role of pollen in the observed adverse associations. Third, this study only used GS% to measure greenspace exposure, which failed to cover scattered vegetation and thus underestimated the greenness cover. The UK Biobank and future research should consider using more sensitive and high-resolution methods for green space assessment. Fourth, 3.47% of the outcome confirmations were based on self-reported data (Table S3), which could introduce recall biases and underestimate the outcome incidence. Fifth, we excluded diseases with a cumulative incidence rate below 1%, which limited the scope of our study. However, this step was taken to reduce bias caused by rare diseases in the statistical analysis and ensure the accuracy and reliability of our research findings. Sixth, additionally incorporating centers as random effects changed a small part of the main findings. This suggested that the associations between greenspace and OAD might be confounded by center-level factors, such as the volume, available equipment, personnel, and patients’ characteristics among different assessment centers. Seventh, causal mediation analysis heavily relies on the correctness of a chosen DAG, the directionality between exposure and mediators, and proportional hazards. Thus, the accuracy of our mediation analysis results has been affected by these factors. Lastly, our study was limited to people living in the UK; thus, the results cannot be directly generalized to other populations with different social and culture contexts and green space characteristics.

## Conclusion

In summary, our study suggests that the associations of greenspace exposures on OAD can be beneficial or detrimental, depending on OAD subtypes. These findings may be useful for policy makers, health professionals, and individuals for transferring greenspace-based area- or individual-level interventions to mitigate the OAD burden.

## Ethical Approval

The UK Biobank obtained ethical approval from the North West MREC (REC reference: 21/NW/0157). All participants provided informed consent for the study to have their records linked to hospital admissions, cancer registries, and death registries.

## Data Availability

Details of how to access UK Biobank data and details of the data release schedule are available from https://www.ukbiobank.ac.uk.

## Supplementary Materials

Supplementary 1 Checklist S1 Fig. S1 Table S1 to S17 Code S1 Supplementary Methods
