# Supplementary material for: Greenspace Exposure and the Incidence of Ocular and Adnexal Diseases: Novel Findings from a Large Prospective Cohort Study
Source: Health Data Sci. 2026 Mar 3;6:0428. doi: 10.34133/hds.0428 (PMC12953923; doi:10.34133/hds.0428)
Supplement: Supplementary 1 — Checklist S1 Fig. S1 Table S1 to S17 Code S1 Supplementary Methods [file hds.0428.f1.docx]

**Supplementary Material**

**Greenspace exposure and the incidence of ocular and adnexal diseases: Novel findings from a large prospective cohort study**

Zhuo-Hao Wang, MD; Xiao-Qi Zhu, MD; Elaine Fuertes, PhD; Hui-Yun Chen, MD; Hui-Ling Qiu, PhD; Gang-Long Zhou, MD; Yu-Ting Xie, MD; Lu Wang, PhD; Jian-Cheng Jiang, MD; Tian-Yu Zhao, MD, PhD; Yu-Zhou Yang, MD; Wen Chen, MD; Joachim Heinrich, MD, PhD; Zhou-Bin Zhang, MD; Bo-Yi Yang, MD, PhD

**Contents**

**Checklist S1.** STROBE Statement—Checklist of items included in “Greenspace exposure and the incidence of ocular and adnexal diseases: Novel findings from a large prospective cohort study”.

**Figure S1.** Directed acyclic graph for the association between greenspace and ocular and adnexal diseases.

**Table S1.** Summary of variables used in the UK Biobank.

**Table S2.** The cumulative incidents/incidence of ocular and adnexal diseases among the study population.

**Table S3.** Sources of report for all types of diseases

**Table S4.** The spearman correlation between greenspace and various mediators

**Table S5.** Results of the proportional hazard test in 300m buffer of greenspace percentage

**Table S6.** Results of the proportional hazard test in 1000m buffer of greenspace percentage

**Table S7.** Results of fitting and tests for adjusted Cox model

**Table S8.** Summary of stratified Cox model (exposure: the percentage of green space within 300m buffer)

**Table S9.** Summary of stratified Cox model (exposure: the percentage of green space within 1000m buffer)

**Table S10.** General characteristics of participants included in and excluded from the current analysis

**Table S11.** The association between the percentage of greenspace within 300m and 1000m buffer and the incidence of ocular and adnexal diseases (including more covariates)

**Table S12.** The association between the percentage of greenspace within 300m and 1000m buffer and the incidence of ocular and adnexal diseases (after excluding incidents within two-years follow-up)

**Table S13.** The association between the percentage of greenspace within 300m and 1000m buffer and the incidence of ocular and adnexal diseases (after excluding multi-OAD patients)

**Table S14.** The association between the percentage of greenspace within 300m and 1000m buffer and the incidence of ocular and adnexal diseases (competing risks model).

**Table S15.** The association between the percentage of greenspace within 300m and 1000m buffer and the incidence of ocular and adnexal diseases after Multiple Imputation by Chained Equations (MICE)

**Table S16.** The association between the percentage of greenspace within 300m and 1000m buffer and the incidence of ocular and adnexal diseases after controlling for the central effect (random term).

**Table S17.** The population attributable fraction of the percentage of greenspace within 300m and 1000m buffer for ocular and adnexal diseases with significant association to greenspace.

**Code S1.** The code for the main data analysis in this study (based on R)

**Supplementary Methods.** Four-way decomposition method

**Checklist S1.** STROBE Statement—Checklist of items included in “Greenspace exposure and the incidence of ocular and adnexal diseases: Novel findings from a large prospective cohort study”

|  | Item No | Recommendation | Section and  paragra |
| --- | --- | --- | --- |
| **Title and abstract** | 1 | (*a*) Indicate the study’s design with a commonly used term in the title or the abstract | Abstract |
|  |  | (*b*) Provide in the abstract an informative and balanced summary of what was done and what was found | Abstract |
| Introduction | | |  |
| Background/rationale | 2 | Explain the scientific background and rationale for the investigation being reported | Introduction, paragraph 1-2 |
| Objectives | 3 | State specific objectives, including any prespecified hypotheses | Introduction, paragraph 2-3 |
| Methods | | |  |
| Study design | 4 | Present key elements of study design early in the paper | Method, paragraph 1 |
| Setting | 5 | Describe the setting, locations, and relevant dates, including periods of recruitment, exposure, follow-up, and data collection | Method, paragraph 1 |
| Participants | 6 | (*a*) Give the eligibility criteria, and the sources and methods of selection of participants. Describe methods of follow-up | Method, paragraph 1-2 & result, paragraph 1 |
|  |  | (*b*) For matched studies, give matching criteria and number of exposed and unexposed | / |
| Variables | 7 | Clearly define all outcomes, exposures, predictors, potential confounders, and effect modifiers. Give diagnostic criteria, if applicable | Method, paragraph 5 |
| Data sources/ measurement | 8* | For each variable of interest, give sources of data and details of methods of assessment (measurement). Describe comparability of assessment methods if there is more than one group | Method, paragraph 1-5 |
| Bias | 9 | Describe any efforts to address potential sources of bias | Statistical analyses, paragraph 3-4 |
| Study size | 10 | Explain how the study size was arrived at | Method, paragraph 1 |
| Quantitative variables | 11 | Explain how quantitative variables were handled in the analyses. If applicable, describe which groupings were chosen and why | Statistical analyses, paragraph 1 |
| Statistical methods | 12 | (*a*) Describe all statistical methods, including those used to control for confounding | Statistical analyses, paragraph 1-5 |
|  |  | (*b*) Describe any methods used to examine subgroups and interactions | / |
|  |  | (*c*) Explain how missing data were addressed | Result, paragraph 1 |
|  |  | (*d*) If applicable, explain how loss to follow-up was addressed | / |
|  |  | (*e*) Describe any sensitivity analyses | Statistical analyses, paragraph 4 |
| Results | | |  |
| Participants | 13* | (a) Report numbers of individuals at each stage of study—eg numbers potentially eligible, examined for eligibility, confirmed eligible, included in the study, completing follow-up, and analysed | Result, paragraph 1 |
|  |  | (b) Give reasons for non-participation at each stage | Result, paragraph 1 & Figure 1 |
|  |  | (c) Consider use of a flow diagram | Result, paragraph 1 & Figure 1 |
| Descriptive data | 14* | (a) Give characteristics of study participants (eg demographic, clinical, social) and information on exposures and potential confounders | Result, paragraph 2 & Table 1 |
|  |  | (b) Indicate number of participants with missing data for each variable of interest | Result, paragraph 1 & Table S10 |
|  |  | (c) Summarise follow-up time (eg, average and total amount) | Result, paragraph 2 |
| Outcome data | 15* | Report numbers of outcome events or summary measures over time | Result, paragraph 2 & Table S2 |
| Main results | 16 | (*a*) Give unadjusted estimates and, if applicable, confounder-adjusted estimates and their precision (eg, 95% confidence interval). Make clear which confounders were adjusted for and why they were included | Method, paragraph 5 & result, paragraph 3 &Figure S1 & Table 2 |
|  |  | (*b*) Report category boundaries when continuous variables were categorized | Result, paragraph 2 & Table 2 |
|  |  | (*c*) If relevant, consider translating estimates of relative risk into absolute risk for a meaningful time period | / |
| Other analyses | 17 | Report other analyses done—eg analyses of subgroups and interactions, and sensitivity analyses | Result, paragraph 4,5 & Table S11-S16 |
| Discussion | | |  |
| Key results | 18 | Summarise key results with reference to study objectives | Discussion, paragraph 1 |
| Limitations | 19 | Discuss limitations of the study, taking into account sources of potential bias or imprecision. Discuss both direction and magnitude of any potential bias | Discussion, paragraph 7 |
| Interpretation | 20 | Give a cautious overall interpretation of results considering objectives, limitations, multiplicity of analyses, results from similar studies, and other relevant evidence | Discussion, paragraph 2-5 |
| Generalisability | 21 | Discuss the generalisability (external validity) of the study results | Discussion, paragraph 6 |
| Other information | | |  |
| Funding | 22 | Give the source of funding and the role of the funders for the present study and, if applicable, for the original study on which the present article is based | Funding |

*Give information separately for exposed and unexposed groups.

Note: An Explanation and Elaboration article discusses each checklist item and gives methodological background and published examples of transparent reporting. The STROBE checklist is best used in conjunction with this article (freely available on the Web sites of PLoS Medicine at http://www.plosmedicine.org/, Annals of Internal Medicine at http://www.annals.org/, and Epidemiology at http://www.epidem.com/). Information on the STROBE Initiative is available at http://www.strobe-statement.org.


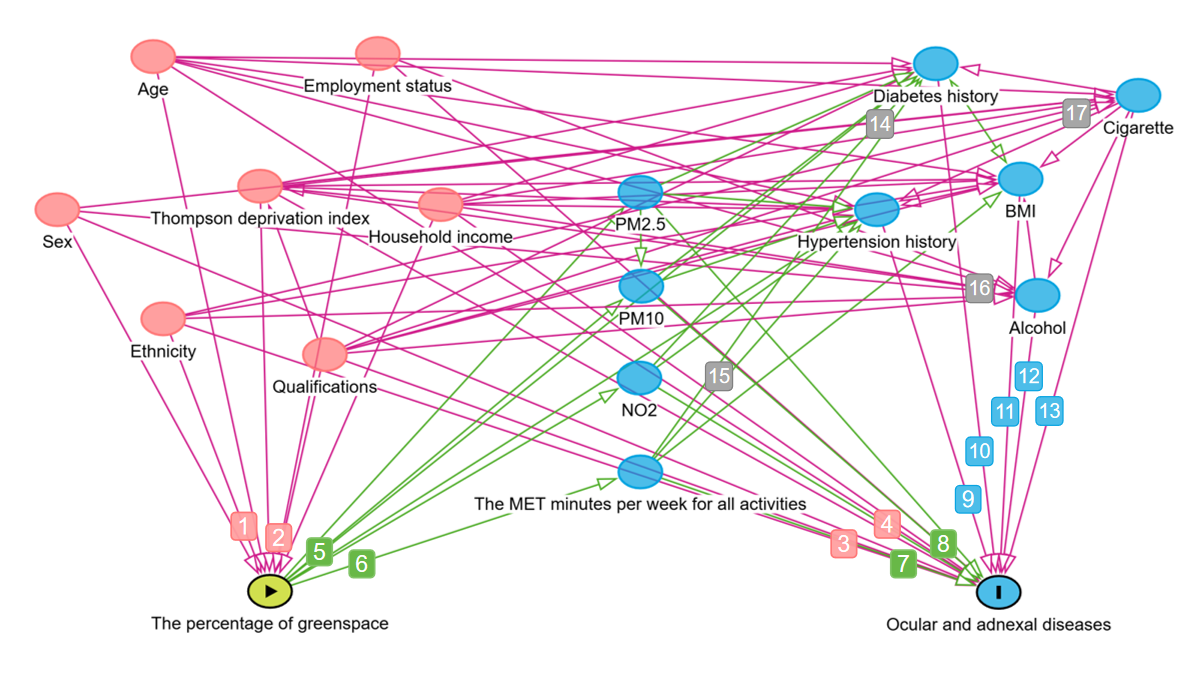


**Figure S1.** Directed acyclic graph for the association between greenspace and ocular and adnexal diseases.

Note: Physical activity was measured by participants’ weekly MET minutes; MET minutes, metabolic equivalent of task minutes are obtained by multiplying the MET value of physical activity by its duration; PM_2.5_, particles with an aerodynamic diameter of ≤ 2.5 μm; PM_10_, particles with an aerodynamic diameter of ≤ 10 μm; NO_2_, nitrogen dioxid.The numbers in the figure are reference numbers.

References for DAG main paths:

[1] Liu M, Timmermans EJ, Wagtendonk A, Meijer P, Grobbee DE, Vaartjes I. Socio-demographic and socio-economic differences in the availability of green space in the Netherlands. *Popul Environ.* 2025;**47(3)**:26. doi:10.1007/s11111-025-00498-3

[2] Zheng L,Kwan MP,Liu Y, et al. How mobility pattern shapes the association between static green space and dynamic green space exposure. *Environ Res.* 2024;**258:**119499. doi:10.1016/j.envres.2024.119499

doi:10.1016/S2468-2667(21)00066-9

[3] Lu C, Miao Y, Yao X, et al. Socioeconomic disparities and green space associated with myopia among Chinese school-aged students: A population-based cohort study. *J Glob Health* 2024;**14**:04140. doi:10.7189/jogh.14.04140

[4] McCormick I,Kim MJ,Hydara A, et al. Socioeconomic position and eye health outcomes: identifying inequality in rapid population-based surveys. *BMJ Open*. 2023;**13 (3)**:e069325. doi:10.1136/bmjopen-2022-069325

Green box: to identify the potential mediating factors

[5] Markevych I, Schoierer J, Hartig T, et al. Exploring pathways linking greenspace to health: Theoretical and methodological guidance. *Environ Res*. 2017; **158**:301–317. https://doi: 10.1016/j.envres.2017.06.028.

[6] Yang BY, Zhao T, Hu LX, et al. Greenspace and human health: An umbrella review. *Innovation (Camb)* 2021; 2:100164. https://doi: 10.1016/j.xinn.2021.100164.

[7] Li SM, Ran AR, Kang MT,et al. Effect of Text Messaging Parents of School-Aged Children on Outdoor Time to Control Myopia: A Randomized Clinical Trial. *JAMA Pediatr* 2022; **176**:1077-1083. https://doi: 10.1001/jamapediatrics.2022.3542.

[8] Wang Z, Yu Y, Ye Y, et al. Associations Between Ambient Air Pollution and Five Common Vision-Threatening Ocular Diseases in Middle-Aged and Older Adults: A Large Prospective Cohort Study. *Am J Ophthalmol.* 2025;**274**:276-285. <https://doi:10.1016/j.ajo.2025.03.009>

Blue box: to identify other important factors

[9] Cheung CY, Biousse V, Keane PA, Schiffrin EL, Wong TY. Hypertensive eye disease. *Nat Rev Dis Primers* 2022; **8**:14. https://doi: 10.1038/s41572-022-00342-0.

[10] Orcutt J, Avakian A, Koepsell TD, Maynard C. Eye disease in veterans with diabetes. *Diabetes Care* 2004; **27**:B50-B53. https://doi: 10.2337/diacare.27.suppl_2.b50.

[11] Ng Yin Ling C,Lim SC,Jonas JB, et al. Obesity and risk of age-related eye diseases: a systematic review of prospective population-based studies. *Int J Obes (Lond)* 2021;**45 (9)**:1863-1885. doi:10.1038/s41366-021-00829-y

[12] Nita M,Grzybowski A. Smoking and Eye Pathologies. A Systemic Review. Part II. Retina Diseases, Uveitis, Optic Neuropathies, Thyroid-Associated Orbitopathy. *Curr Pharm Des* 2017;**23 (4)**:639-654. doi:10.2174/1381612823666170111095723

[13] Wang S,Wang JJ,Wong TY. Alcohol and eye diseases. *Surv Ophthalmol* 2008**;53 (5)**:512-25. doi:10.1016/j.survophthal.2008.06.003

Grey box: references for the paths between other variables

[14] Howell NA,Tu JV,Moineddin R, et al. Interaction between neighborhood walkability and traffic-related air pollution on hypertension and diabetes: The CANHEART cohort. *Environ Int* 2019;**132**:104799. doi:10.1016/j.envint.2019.04.070

[15] Balducci S,Haxhi J,Sacchetti M, et al. Relationships of Changes in Physical Activity and Sedentary Behavior With Changes in Physical Fitness and Cardiometabolic Risk Profile in Individuals With Type 2 Diabetes: The Italian Diabetes and Exercise Study 2 (IDES_2). *Diabetes Care* 2022;**45 (1)**:213-221. doi:10.2337/dc21-1505

[16] Auguste A,Dugas J,Menvielle G, et al. Social distribution of tobacco smoking, alcohol drinking and obesity in the French West Indies. *BMC Public Health*. 2019;**19 (1)**:1424. doi:10.1186/s12889-019-7802-1

[17] Mahdaviazad H,Foroutan R,Masoompour SM. Prevalence of tobacco smoking and its socioeconomic determinants: Tobacco smoking and its determinants. *Clin Respir J.* 2022;**16 (3)**:208-215. doi:10.1111/crj.13470

**Table S1.** Summary of variables used in the UK Biobank

|  | **Category** | **Category/Field ID description** |
| --- | --- | --- |
| Body mass index | 100010 | 21001 |
| Date of attending assessment centre | 100024 | 53 |
| Health and medical history-Eyesight | 100036 | 100041 |
| Ethnic background | 100065 | 21000 |
| Age at recruitment | 100094 | 21022 |
| [Sex](https://biobank.ctsu.ox.ac.uk/crystal/field.cgi?id=31) | 100094 | 31 |
| Thompson deprivation index | 100094 | 22189 |
| Qualifications | 100062 | 6138 |
| Employment status | 100064 | 6142 |
| Average total household income before tax | 100066 | 738 |
| Current somking | 100058 | 20116 |
| Alcohol drinker status | 100051 | 20117 |
| First occurrences-Endocrine, nutritional and metabolic disorders | 1712 | 2404 |
| First occurrences-[Eye and adnexa disorders](https://biobank.ctsu.ox.ac.uk/crystal/label.cgi?id=2407) | 1712 | 2407 |
| First occurrences-Circulatory system disorders | 1712 | 2409 |
| [Summed MET minutes per week for all activity](https://biobank.ctsu.ox.ac.uk/crystal/field.cgi?id=22040) | 54 | 22040 |
| [Nitrogen dioxide air pollution; 2010](https://biobank.ctsu.ox.ac.uk/crystal/field.cgi?id=24003) | 711 | 24003 |
| [Particulate matter air pollution (pm10); 2010](https://biobank.ctsu.ox.ac.uk/crystal/field.cgi?id=24005) | 711 | 24005 |
| [Particulate matter air pollution (pm2.5); 2010](https://biobank.ctsu.ox.ac.uk/crystal/field.cgi?id=24006) | 711 | 24006 |
| [Greenspace percentage, buffer 1000m](https://biobank.ctsu.ox.ac.uk/crystal/field.cgi?id=24503) | 711 | 24500 |
| [Greenspace percentage, buffer 300m](https://biobank.ctsu.ox.ac.uk/crystal/field.cgi?id=24503) | 711 | 24503 |

**Table S2.** The cumulative incidents/incidence of ocular and adnexal diseases among the study population

| **Diseases categories (ICD-10 code)** | **Cumulative incidents (Cumulative incidence)** |
| --- | --- |
| Total ocular and adnexal diseases (H00-H59) | 43114 (17.85%) |
| Disorders of eyelid, lacrimal system and orbit (H00-H06) | 9293 (3.84%) |
| Disorders of conjunctiva (H10-H13) | 3936 (1.63%) |
| Disorders of sclera, cornea, iris and ciliary body (H15-H22) | 2643 (1.09%) |
| Disorders of lens (H25-H28) | 22787 (9.43%) |
| Disorders of choroid and retina (H30-H36) | 8160 (3.38%) |
| Glaucoma (H40-H42) | 5165 (2.14%) |
| Disorders of vitreous body and globe (H43-H45) | 3397 (1.41%) |
| Disorders of optic nerve and visual pathways (H46-H48) | 481 (0.20%) |
| Disorders of ocular muscles, binocular movement, accommodation and refraction (H49-H52) | 4757 (1.97%) |
| Visual disturbances and blindness (H53-H54) | 4512 (1.87%) |
| Other disorders of eye and adnexa (H55-H59) | 1781 (0.74%) |

Note: The longitudinal study population consists of 241,576 individual.

**Table S3.** Sources of report for all types of diseases

| **Diseases categories (ICD-10 code)** | **Number of incidents (n)** | | | | | | | |
| --- | --- | --- | --- | --- | --- | --- | --- | --- |
|  | **Primary care only** | **Primary care and other source (s)** | **Hospital admissions data only** | **Hospital admissions data and other source (s)** | **Self-report only** | **Self-report and other source (s)** | **Death register only** | **Death register and other source (s)** |
| Hordeolum and chalazion (H00) | 1491 | 62 | 507 | 4 | 0 | 0 | 0 | 0 |
| Other inflammation of eyelid (H01) | 331 | 6 | 538 | 1 | 73 | 0 | 0 | 0 |
| Other disorders of eyelid (H02) | 398 | 101 | 2915 | 14 | 75 | 3 | 0 | 0 |
| Disorders of eyelid in diseases classified elsewhere (H03) | 1 | 0 | 24 | 0 | 0 | 0 | 0 | 0 |
| Disorders of lachrymal system (H04) | 1848 | 117 | 1396 | 7 | 179 | 8 | 0 | 0 |
| Disorders of orbit (H05) | 108 | 8 | 152 | 3 | 0 | 0 | 0 | 0 |
| Disorders of lachrymal system and orbit in diseases classified elsewhere (H06) | 0 | 0 | 51 | 0 | 0 | 0 | 0 | 0 |
| Conjunctivitis (H10) | 1979 | 10 | 217 | 4 | 17 | 0 | 0 | 0 |
| Other disorders of conjunctiva (H11) | 1192 | 9 | 546 | 3 | 0 | 0 | 0 | 0 |
| Disorders of conjunctiva in diseases classified elsewhere (H13) | 23 | 0 | 26 | 0 | 0 | 0 | 0 | 0 |
| Disorders of sclera (H15) | 144 | 0 | 52 | 0 | 0 | 0 | 0 | 0 |
| Keratitis (H16) | 83 | 1 | 151 | 3 | 0 | 0 | 0 | 0 |
| Corneal scars and opacities (H17) | 17 | 1 | 107 | 1 | 0 | 0 | 0 | 0 |
| Other disorders of cornea (H18) | 132 | 26 | 1313 | 3 | 0 | 0 | 0 | 0 |
| Disorders of sclera and cornea in diseases classified elsewhere (H19) | 7 | 0 | 44 | 0 | 0 | 0 | 0 | 0 |
| Iridocyclitis (H20) | 127 | 6 | 196 | 2 | 9 | 1 | 0 | 0 |
| Other disorders of iris and ciliary body (H21) | 26 | 2 | 392 | 1 | 0 | 0 | 0 | 0 |
| Disorders of iris and ciliary body in diseases classified elsewhere (H22) | 0 | 0 | 21 | 0 | 0 | 0 | 0 | 0 |
| Senile cataract (H25) | 30 | 35 | 13066 | 3 | 0 | 0 | 0 | 0 |
| Other cataract (H26) | 119 | 124 | 12623 | 121 | 1216 | 401 | 0 | 0 |
| Other disorders of lens (H27) | 1 | 0 | 208 | 0 | 0 | 0 | 0 | 0 |
| Cataract and other disorders of lens in diseases classified elsewhere (H28) | 0 | 0 | 15 | 0 | 0 | 0 | 0 | 0 |
| Chorioretinal inflammation (H30) | 11 | 2 | 15 | 2 | 0 | 0 | 0 | 0 |
| Other disorders of choroid (H31) | 35 | 3 | 174 | 1 | 0 | 0 | 0 | 0 |
| Chorioretinal disorders in diseases classified elsewhere (H32) | 1 | 0 | 5 | 0 | 0 | 0 | 0 | 0 |
| Retinal detachments and breaks (H33) | 81 | 54 | 1611 | 63 | 45 | 26 | 0 | 0 |
| Retinal vascular occlusions (H34) | 157 | 62 | 426 | 8 | 24 | 3 | 0 | 0 |
| Other retinal disorders (H35) | 679 | 238 | 4058 | 47 | 111 | 33 | 1 | 0 |
| Retinal disorders in diseases classified elsewhere (H36) | 1 | 0 | 1332 | 0 | 49 | 12 | 0 | 0 |
| Glaucoma (H40) | 773 | 365 | 3645 | 68 | 171 | 143 | 0 | 0 |
| Glaucoma in diseases classified elsewhere (H42) | 0 | 0 | 0 | 0 | 0 | 0 | 0 | 0 |
| Disorders of vitreous body (H43) | 1503 | 46 | 1403 | 4 | 0 | 0 | 0 | 0 |
| Disorders of globe (H44) | 177 | 1 | 327 | 1 | 0 | 0 | 0 | 0 |
| Disorders of vitreous body and globe in diseases classified elsewhere (H45) | 0 | 0 | 5 | 0 | 0 | 0 | 0 | 0 |
| Optic neuritis (H46) | 4 | 1 | 49 | 0 | 5 | 0 | 0 | 0 |
| Other disorders of optic 2nd nerve and visual pathways (H47) | 126 | 5 | 294 | 4 | 0 | 0 | 0 | 0 |
| Disorders of optic 2nd nerve and visual pathways in diseases classified elsewhere (H48) | 0 | 0 | 3 | 0 | 0 | 0 | 0 | 0 |
| Paralytic strabismus (H49) | 25 | 11 | 222 | 6 | 0 | 0 | 0 | 0 |
| Other strabismus (H50) | 28 | 11 | 414 | 3 | 0 | 0 | 0 | 0 |
| Other disorders of binocular movement (H51) | 18 | 0 | 26 | 0 | 0 | 0 | 0 | 0 |
| Disorders of refraction and accommodation (H52) | 131 | 41 | 3918 | 3 | 0 | 0 | 0 | 0 |
| Visual disturbances (H53) | 704 | 38 | 2810 | 7 | 0 | 0 | 0 | 0 |
| Blindness and low vision (H54) | 196 | 7 | 865 | 5 | 0 | 0 | 0 | 0 |
| Nystagmus and other irregular eye movements (H55) | 4 | 1 | 227 | 0 | 0 | 0 | 0 | 0 |
| Other disorders of eye and adnexa (H57) | 909 | 3 | 431 | 0 | 0 | 0 | 0 | 0 |
| Other disorders of eye and adnexa in diseases classified elsewhere (H58) | 0 | 0 | 83 | 0 | 0 | 0 | 0 | 0 |
| Postprocedural disorders of eye and adnexanot elsewhere classified (H59) | 1 | 0 | 143 | 0 | 0 | 0 | 0 | 0 |

Note: The outcome ascertaining procedure in UK Biobank can be found in the following link: “<https://biobank.ctsu.ox.ac.uk/crystal/ukb/docs/first_occurrences_outcomes.pdf>”

**Table S4.** The spearman correlation between greenspace and various mediators

|  | **GS% within 300m buffer** | **GS% within 1000m buffer** | **PM_2.5_** | **PM_10_** | **NO_2_** | **Weekly MET minutes** |
| --- | --- | --- | --- | --- | --- | --- |
| **GS% within 300m buffer** | 1.000 | 0.826^*^ | -0.685^*^ | -0.421^*^ | -0.675^*^ | 0.010^*^ |
| **GS% within 1000m buffer** | - | 1.000 | -0.701^*^ | -0.408^*^ | -0.778^*^ | 0.010^*^ |
| **PM_2.5_** | - | - | 1.000 | 0.577^*^ | 0.890^*^ | -0.007^*^ |
| **PM_10_** | - | - | - | 1.000 | 0.531^*^ | -0.004 |
| **NO_2_** | - | - | - | - | 1.000 | -0.007^*^ |
| **Weekly MET minutes** | - | - | - | - | - | 1.000 |

Note: GS%, percentage of greenspace; MET minutes, weekly metabolic equivalent of task minutes.

^*^Spearman correlation: *p*-value <0.01.

**Table S5.** Results of the proportional hazard test in 300-m buffer of greenspace percentage

| **OAD subtype**  **(ICD-10 code)** | **Variables [Chisq (P-value)]** | | | | | | | | |
| --- | --- | --- | --- | --- | --- | --- | --- | --- | --- |
|  | **GS%300m_Q (df=3)** | **Age**  **(df=1)** | **Sex**  **(df=1)** | **Ethnicity**  **(df=1)** | **Thompson deprivation index**  **(df=4)** | **Household income**  **(df=3)** | **Qualifications**  **(df=4)** | **Employment status**  **(df=2)** | **Global**  **(df=3 or 19)** |
| H00-H06 | 0.814 (0.85) | - | - | - | - | - | - | - | 0.814 (0.85) |
|  | 0.861 (0.83) | 134.799 (<0.001) | 19.465 (<0.001) | 2.294 (0.13) | 4.214 (0.38) | 3.183 (0.36) | 9.496 (0.05) | 37.219 (<0.001) | 164.904 (<0.001) |
| H10-H13 | 8.18 (0.042) | - | - | - | - | - | - | - | 8.18 (0.042) |
|  | 8.24 (0.041) | 16.78 (<0.001) | 5.56 (0.018) | 2.02 (0.155) | 11.06 (0.026) | 2.08 (0.557) | 10.07 (0.039) | 4.22 (0.121) | 57.60 (<0.001) |
| H15-H22 | 2.64 (0.45) | - | - | - | - | - | - | - | 2.64 (0.45) |
|  | 2.584 (0.46) | 233.497 (<0.001) | 0.559 (0.455) | 2.375 (0.123) | 10.498 (0.033) | 53.433 (<0.001) | 32.397 (<0.001) | 109.265 (<0.001) | 264.159 (<0.001) |
| H25-H28 | 5.49 (0.14) | - | - | - | - | - | - | - | 5.49 (0.14) |
|  | 4.564 (0.207) | 13.423 (<0.001) | 23.556 (<0.001) | 0.515 (0.473) | 4.217 (0.377) | 3.518 (0.318) | 6.941 (0.139) | 11.934 (0.003) | 66.57 (<0.001) |
| H30-H36 | 2.57 (0.46) | - | - | - | - | - | - | - | 2.57 (0.46) |
|  | 2.627 (0.453) | 21.173 (<0.001) | 14.339 (<0.001) | 0.171 (0.680) | 12.892 (0.012) | 12.995 (0.005) | 15.72 (0.003) | 14.529 (<0.001) | 68.209 (<0.001) |
| H40-H42 | 11.3 (0.01) | - | - | - | - | - | - | - | 11.3 (0.01) |
|  | 11.610 (0.009) | 22.447 (<0.001) | 0.189 (0.663) | 0.004 (0.948) | 8.417 (0.077) | 10.258 (0.016) | 9.763 (0.045) | 3.820 (0.148) | 47.978 (<0.001) |
| H43-H45 | 22.8 (<0.001) | - | - | - | - | - | - | - | 22.8 (<0.001) |
|  | 22.925 (<0.001) | 0.225 (0.635) | 9.062 (0.003) | 1.209 (0.272) | 16.767 (0.002) | 1.505 (0.681) | 13.347 (0.010) | 1.143 (0.565) | 58.813 (<0.001) |
| H49-H52 | 5.64 (0.13) | - | - | - | - | - | - | - | 5.64 (0.13) |
|  | 5.589 (0.13) | 89.563 (<0.001) | 0.542 (0.46) | 2.076 (0.15) | 5.073 (0.28) | 49.235 (<0.001) | 44.59 (<0.001) | 33.007 (<0.001) | 138.021 (<0.001) |
| H53-H54 | 6.02 (0.11) | - | - | - | - | - | - | - | 6.02 (0.11) |
|  | 6.182 (0.103) | 46.957 (<0.001) | 0.540 (0.463) | 0.022 (0.883) | 3.840 (0.428) | 12.239 (0.007) | 17.394 (0.002) | 23.334 (<0.001) | 67.490 (<0.001) |

Note: GS%300m_Q; The percentage of green space within the 300-m buffer (categorical variable); Disorders of eyelid, lacrimal system and orbit (H00-H06); Disorders of conjunctiva (H10-H13); Disorders of sclera, cornea, iris and ciliary body (H15-H22); Disorders of lens (H25-H28); Disorders of choroid and retina (H30-H36); Glaucoma (H40-H42); Disorders of vitreous body and globe (H43-H45); Disorders of ocular muscles, binocular movement, accommodation and refraction (H49-H52); Visual disturbances and blindness (H53-H54)

**Table S6.** Results of the proportional hazard test in 1000-m buffer of greenspace percentage

| **OAD subtype**  **(ICD-10 code)** | **Variables [Chisq (P-value)]** | | | | | | | | |
| --- | --- | --- | --- | --- | --- | --- | --- | --- | --- |
|  | **GS%1000m_Q (df=3)** | **Age**  **(df=1)** | **Sex**  **(df=1)** | **Ethnicity**  **(df=1)** | **Thompson deprivation index**  **(df=4)** | **Household income**  **(df=3)** | **Qualifications**  **(df=4)** | **Employment status**  **(df=2)** | **Global**  **(df=3 or 19)** |
| H00-H06 | 0.897 (0.83) | - | - | - | - | - | - | - | 0.897 (0.83) |
|  | 0.881 (0.83) | 134.883 (<0.001) | 19.481 (<0.001) | 2.296 (0.13) | 4.199 (0.38) | 3.197 (0.36) | 9.509 (0.05) | 37.257 (<0.001) | 166.071 (<0.001) |
| H10-H13 | 10.2 (0.017) | - | - | - | - | - | - | - | 10.2 (0.017) |
|  | 10.21 (0.017) | 16.78 (<0.001) | 5.57 (0.018) | 2.02 (0.155) | 11.06 (0.026) | 2.07 (0.557) | 10.07 (0.039) | 4.22 (0.121) | 59.58 (<0.001) |
| H15-H22 | 1.73 (0.63) | - | - | - | - | - | - | - | 1.73 (0.63) |
|  | 1.81 (0.613) | 233.43 (<0.001) | 0.561 (0.454) | 2.376 (0.123) | 10.485 (0.033) | 53.451 (<0.001) | 32.405 (<0.001) | 109.325 (<0.001) | 264.161 (<0.001) |
| H25-H28 | 6.42 (0.093) | - | - | - | - | - | - | - | 6.42 (0.093) |
|  | 5.039 (0.169) | 13.393 (<0.001) | 23.571 (<0.001) | 0.517 (0.472) | 4.221 (0.377) | 3.544 (0.315) | 6.946 (0.139) | 11.901 (0.003) | 67.267 (<0.001) |
| H30-H36 | 2.2 (0.53) | - | - | - | - | - | - | - | 2.2 (0.53) |
|  | 2.452 (0.484) | 21.172 (<0.001) | 14.334 (<0.001) | 0.171 (0.680) | 12.883 (0.012) | 12.995 (0.005) | 15.736 (0.003) | 14.534 (<0.001) | 66.382 (<0.001) |
| H40-H42 | 14.1 (0.003) | - | - | - | - | - | - | - | 14.1 (0.003) |
|  | 14.386 (0.002) | 22.452 (<0.001) | 0.189 (0.664) | 0.004 (0.948) | 8.417 (0.077) | 10.259 (0.016) | 9.768 (0.045) | 3.822 (0.148) | 49.968 (<0.001) |
| H43-H45 | 41.3 (<0.001) | - | - | - | - | - | - | - | 41.3 (<0.001) |
|  | 41.519 (<0.001) | 0.225 (0.635) | 9.067 (0.003) | 1.211 (0.271) | 16.774 (0.002) | 1.502 (0.682) | 13.357 (0.010) | 1.141 (0.565) | 75.074 (<0.001) |
| H49-H52 | 21.5 (<0.001) | - | - | - | - | - | - | - | 21.5 (<0.001) |
|  | 21.47 (<0.001) | 89.497 (<0.001) | 0.543 (0.46) | 2.08 (0.15) | 5.077 (0.28) | 49.252 (<0.001) | 44.627 (<0.001) | 33.01 (<0.001) | 148.659 (<0.001) |
| H53-H54 | 2.07 (0.56) | - | - | - | - | - | - | - | 2.07 (0.56) |
|  | 2.270 (0.518) | 46.942 (<0.001) | 0.540 (0.463) | 0.022 (0.883) | 3.840 (0.428) | 12.240 (0.007) | 17.398 (0.002) | 23.329 (<0.001) | 63.495 (<0.001) |

Note: GS%1000m_Q; The percentage of green space within the 1000-m buffer (categorical variable); Disorders of eyelid, lacrimal system and orbit (H00-H06); Disorders of conjunctiva (H10-H13); Disorders of sclera, cornea, iris and ciliary body (H15-H22); Disorders of lens (H25-H28); Disorders of choroid and retina (H30-H36); Glaucoma (H40-H42); Disorders of vitreous body and globe (H43-H45); Disorders of ocular muscles, binocular movement, accommodation and refraction (H49-H52); Visual disturbances and blindness (H53-H54)

**Table S7.** Results of fitting and tests for adjusted Cox model

| **OAD subtype**  **(ICD-10 code)** | | **Test methods [Statistics (*P*-value, df)]** | | | |
| --- | --- | --- | --- | --- | --- |
|  |  | **Concordance** | **Likelihood ratio test** | **Wald test** | **Score (logrank) test** |
| H00-H06 | GS%_300m | 0.533 (se=0.003) | 117 (<0.001, 15) | 117.4 (<0.001, 15) | 117.7 (<0.001, 15) |
|  | GS%_1000m | 0.516 (se = 0.004) | 13.22 (0.004, 15) | 13.27 (0.004, 15) | 13.28 (0.004, 15) |
| H10-H13 | GS%_300m | 0.536 (se = 0.005) | 55.47 (<0.001, 10) | 55.98 (<0.001, 10) | 56.11 (<0.001, 10) |
|  | GS%_1000m | 0.538 (se = 0.005) | 66.56 (<0.001, 10) | 66.6 (<0.001, 10) | 66.77 (<0.001, 10) (<0.001, 10) |
| H15-H22 | GS%_300m | 0.518 (se = 0.007) | 7.39 (0.200, 5) | 7.4 (0.200, 5) | 7.4 (0.200, 5) |
|  | GS%_1000m | 0.525 (se = 0.007) | 12.51 (0.030, 5) | 12.56 (0.030, 5) | 12.57 (0.030, 5) |
| H25-H28 | GS%_300m | 0.535 (se = 0.002) | 349.4 (<0.001, 15) | 350.4 (<0.001, 15) | 351.7 (<0.001, 15) |
|  | GS%_1000m | 0.535 (se = 0.002) | 360.5 (<0.001, 15) | 360.9 (<0.001, 15) | 362.2 (<0.001, 15) |
| H30-H36 | GS%_300m | 0.510 (se = 0.004) | 7.07 (0.100, 4) | 7.01 (0.100, 4) | 7.01 (0.100, 4) |
|  | GS%_1000m | 0.512 (se = 0.004) | 7.46 (0.100, 4) | 7.4 (0.100, 4) | 7.4 (0.100, 4) |
| H40-H42 | GS%_300m | 0.519 (se = 0.005) | 40.21 (<0.001, 12) | 41.41 (<0.001, 12) | 41.5 (<0.001, 12) |
|  | GS%_1000m | 0.519 (se = 0.005) | 40.25 (<0.001, 12) | 41.45 (<0.001, 12) | 41.55 (<0.001, 12) |
| H43-H45 | GS%_300m | 0.659 (se = 0.005) | 934.5 (<0.001, 11) | 849.5 (<0.001, 11) | 891.0 (<0.001, 11) |
|  | GS%_1000m | 0.658 (se = 0.005) | 923.6 (<0.001, 11) | 839.1 (<0.001, 11) | 880.6 (<0.001, 11) |
| H49-H52 | GS%_300m | 0.540 (se = 0.005) | 110.4 (<0.001, 9) | 110.7 (<0.001, 9) | 111.1 (<0.001, 9) |
|  | GS%_1000m | 0.542 (se = 0.006) | 118.8 (<0.001, 10) | 119.1 (<0.001, 10) | 119.6 (<0.001, 10) |
| H53-H54 | GS%_300m | 0.535 (se = 0.006) | 62.81 (<0.001, 9) | 63.44 (<0.001, 9) | 63.66 (<0.001, 9) |
|  | GS%_1000m | 0.535 (se = 0.006) | 61.14 (<0.001, 9) | 61.8 (<0.001, 9) | 62.02 (<0.001, 9) |

Note: GS%_300m; The percentage of green space within 300-m buffer; GS%_1000m; The percentage of green space within 1000-m buffer; SE, standard error; Disorders of eyelid, lacrimal system and orbit (H00-H06); Disorders of conjunctiva (H10-H13); Disorders of sclera, cornea, iris and ciliary body (H15-H22); Disorders of lens (H25-H28); Disorders of choroid and retina (H30-H36); Glaucoma (H40-H42); Disorders of vitreous body and globe (H43-H45); Disorders of ocular muscles, binocular movement, accommodation and refraction (H49-H52); Visual disturbances and blindness (H53-H54)

**Table S8.** Summary of stratified Cox model (exposure: the percentage of green space within 300-m buffer)

| **Predictor variables** | **Hazard ratio (95% confidence interval)** | | | | | | | | |
| --- | --- | --- | --- | --- | --- | --- | --- | --- | --- |
|  | **H00-H06** | **H10-H13** | **H15-H22** | **H25-H28** | **H30-H36** | **H40-H42** | **H43-H45** | **H49-H52** | **H53-H54** |
| Age | / | / | / | / | / | / | 1.079 (1.072, 1.085)* | / | / |
| Sex |  | | | | | | | | |
| Female | Ref. | | | | | | | | |
| Male | / | / | 0.912 (0.841, 0.988)* | / | / | 1.008 (0.954, 1.066) | / | 0.778 (0.733, 0.825)* | 1.079 (1.016, 1.146)* |
| Ethnic |  | | | | | | | | |
| Non-White | Ref. | | | | | | | | |
| White | 1.007 (0.922, 1.101) | 1.053 (0.915, 1.211) | 0.968 (0.818, 1.146) | 1.019 (0.962, 1.078) | 0.971 (0.880, 1.070) | 0.993 (0.882, 1.118) | 0.986 (0.852, 1.141) | 1.021 (0.900, 1.157) | 1.031 (0.906, 1.173) |
| TDI |  | | | | | | | | |
| Low Deprivation | Ref. | | | | | | | | |
| Mildly Deprivation | 1.023 (0.959, 1.091) | / | / | 0.965 (0.926, 1.006) | / | 0.937 (0.859, 1.022) | / | 0.983 (0.897, 1.078) | 1.094 (0.991, 1.207) |
| Moderately Deprivation | 1.025 (0.961, 1.094) | / | / | 0.992 (0.951, 1.034) | / | 0.982 (0.901, 1.071) | / | 1.028 (0.938, 1.127) | 1.101 (0.998, 1.216) |
| Highly Deprivation | 1.042 (0.975, 1.113) | / | / | 1.039 (0.996, 1.083) | / | 0.977 (0.893, 1.068) | / | 1.098 (1.001, 1.205)* | 1.219 (1.104, 1.346)* |
| Severely Deprivation | 1.081 (1.009, 1.159)* | / | / | 1.184 (1.134, 1.237)* | / | 1.157 (1.056, 1.267)* | / | 1.258 (1.143, 1.385)* | 1.398 (1.265, 1.546)* |
| Employment status |  | | | | | | | | |
| Employed | Ref. | | | | | | | | |
| Retired | / | 1.063 (0.965, 1.170) | / | / | / | 1.013 (0.937, 1.095) | 0.835 (0.762, 0.915)* | / | / |
| Unemployed, home maker, or other | / | 1.042 (0.919, 1.182) | / | / | / | 1.230 (1.095, 1.381)* | 0.922 (0.794, 1.071) | / | / |
| Household income |  | | | | | | | | |
| <£18 000 | Ref. | | | | | | | | |
| £18 000–£30 999 | 0.939 (0.887, 0.994)* | 0.849 (0.776, 0.928)* | / | 0.942 (0.910, 0.975)* | / | / | 1.015 (0.923, 1.115) | / | / |
| £31 000–£51 999 | 0.884 (0.829, 0.943)* | 0.800 (0.724, 0.882)* | / | 0.879 (0.844, 0.915)* | / | / | 0.928 (0.834, 1.032) | / | / |
| ≥£52 000 | 0.829 (0.772, 0.891)* | 0.723 (0.648, 0.806)* | / | 0.762 (0.726, 0.800)* | / | / | 0.893 (0.794, 1.005) | / | / |
| Qualifications |  | | | | | | | | |
| None | Ref. | | | | | | | | |
| O levels, GCSEs, or CSEs | 0.926 (0.873, 0.983)* | / | / | 1.008 (0.973, 1.044) | / | / | / | / | / |
| A levels or AS levels | 0.945 (0.844, 1.058) | / | / | 1.026 (0.955, 1.102) | / | / | / | / | / |
| NVQ, HND, HNC, or other professional qualification | 1.041 (0.968, 1.119) | / | / | 1.021 (0.976, 1.067) | / | / | / | / | / |
| College or university degree | 0.867 (0.801, 0.938)* | / | / | 1.032 (0.984, 1.083) | / | / | / | / | / |

Note: TDI, Indeices of thompson deprivation; Disorders of eyelid, lacrimal system and orbit (H00-H06); Disorders of conjunctiva (H10-H13); Disorders of sclera, cornea, iris and ciliary body (H15-H22); Disorders of lens (H25-H28); Disorders of choroid and retina (H30-H36); Glaucoma (H40-H42); Disorders of vitreous body and globe (H43-H45); Disorders of ocular muscles, binocular movement, accommodation and refraction (H49-H52); Visual disturbances and blindness (H53-H54). Qualifications: O levels, Ordinary levels;GCSE, General Certificate of Secondary Education; CSE, Certificate of Secondary Education, A levels, Advanced levels; AS levels, Advanced Subsidiary levels; NVQ, National Vocational Qualification; HND, Higher National Diploma; HNC, Higher National Certificate; **P*-value <0.05.

**Table S9.** Summary of stratified Cox model (exposure: the percentage of green space within 1000-m buffer)

| **Predictor variables** | **Hazard ratio (95% confidence interval)** | | | | | | | | | | | | | | | | |
| --- | --- | --- | --- | --- | --- | --- | --- | --- | --- | --- | --- | --- | --- | --- | --- | --- | --- |
|  | **H00-H06** | **H10-H13** | | **H15-H22** | | **H25-H28** | | **H30-H36** | | **H40-H42** | | **H43-H45** | | **H49-H52** | | **H53-H54** | |
| Age | / | | / | | / | | / | | / | | / | | 1.079 (1.072, 1.085)* | | / | | / |
| Sex |  | | | | | | | | | | | | | | | | |
| Female | Ref. | | | | | | | | | | | | | | | | |
| Male | / | | / | | 0.912 (0.842, 0.989)* | | / | | / | | 1.008 (0.954, 1.066) | | / | | 0.778 (0.733, 0.826)* | | 1.080 (1.016, 1.147)* |
| Ethnic |  | | | | | | | | | | | | | | | | |
| Non-White | Ref. | | | | | | | | | | | | | | | | |
| White | 1.007 (0.921, 1.101) | | 1.053 (0.915, 1.211) | | 0.967 (0.818, 1.145) | | 1.018 (0.961, 1.078) | | 0.971 (0.880, 1.070) | | 0.993 (0.881, 1.118) | | 0.987 (0.853, 1.142) | | 1.021 (0.900, 1.158) | | 1.031 (0.906, 1.174) |
| TDI |  | | | | | | | | | | | | | | | | |
| Low Deprivation | Ref. | | | | | | | | | | | | | | | | |
| Mildly Deprivation | 1.029 (0.965, 1.098) | | / | | / | | 0.966 (0.927, 1.007) | | / | | 0.938 (0.860, 1.023) | | / | | 0.980 (0.894, 1.074) | | 1.092 (0.990, 1.206) |
| Moderately Deprivation | 1.038 (0.972, 1.108) | | / | | / | | 0.990 (0.949, 1.032) | | / | | 0.986 (0.904, 1.075) | | / | | 1.014 (0.925, 1.112) | | 1.099 (0.995, 1.213) |
| Highly Deprivation | 1.067 (0.998, 1.141) | | / | | / | | 1.035 (0.992, 1.080) | | / | | 0.985 (0.900, 1.078) | | / | | 1.067 (0.972, 1.173) | | 1.216 (1.100, 1.344)* |
| Severely Deprivation | 1.123 (1.046, 1.206)* | | / | | / | | 1.178 (1.126, 1.232)* | | / | | 1.173 (1.069, 1.289)* | | / | | 1.203 (1.090, 1.328)* | | 1.392 (1.256, 1.543)* |
| Employment status |  | | | | | | | | | | | | | | | | |
| Employed | Ref. | | | | | | | | | | | | | | | | |
| Retired | / | | 1.062 (0.964, 1.169) | | / | | / | | / | | 1.012 (0.936, 1.094) | | 0.836 (0.763, 0.916)* | | / | | / |
| Unemployed, home maker, or other | / | | 1.043 (0.919, 1.183) | | / | | / | | / | | 1.229 (1.094, 1.380)* | | 0.922 (0.794, 1.071) | | / | | / |
| Household income |  | | | | | | | | | | | | | | | | |
| <£18 000 | Ref. | | | | | | | | | | | | | | | | |
| £18 000–£30 999 | 0.939 (0.887, 0.994)* | | 0.849 (0.776, 0.929)* | | / | | 0.942 (0.910, 0.975)* | | / | | / | | 1.016 (0.924, 1.117) | | / | | / |
| £31 000–£51 999 | 0.884 (0.829, 0.943)* | | 0.800 (0.725, 0.883)* | | / | | 0.879 (0.844, 0.916)* | | / | | / | | 0.929 (0.836, 1.033) | | / | | / |
| ≥£52 000 | 0.831 (0.774, 0.893)* | | 0.724 (0.650, 0.808)* | | / | | 0.763 (0.727, 0.801)* | | / | | / | | 0.897 (0.797, 1.009) | | / | | / |
| Qualifications |  | | | | | | | | | | | | | | | | |
| None | Ref. | | | | | | | | | | | | | | | | |
| O levels, GCSEs, or CSEs | 0.928 (0.875, 0.985)* | | / | | / | | 1.009 (0.973, 1.045) | | / | | / | | / | | / | | / |
| A levels or AS levels | 0.949 (0.848, 1.063) | | / | | / | | 1.027 (0.956, 1.103) | | / | | / | | / | | / | | / |
| NVQ, HND, HNC, or other professional qualification | 1.042 (0.969, 1.120) | | / | | / | | 1.022 (0.977, 1.068) | | / | | / | | / | | / | | / |
| College or university degree | 0.873 (0.806, 0.944)* | | / | | / | | 1.033 (0.985, 1.084) | | / | | / | | / | | / | | / |

Note: TDI, Indeices of thompson deprivation; Disorders of eyelid, lacrimal system and orbit (H00-H06); Disorders of conjunctiva (H10-H13); Disorders of sclera, cornea, iris and ciliary body (H15-H22); Disorders of lens (H25-H28); Disorders of choroid and retina (H30-H36); Glaucoma (H40-H42); Disorders of vitreous body and globe (H43-H45); Disorders of ocular muscles, binocular movement, accommodation and refraction (H49-H52); Visual disturbances and blindness (H53-H54). Qualifications: O levels, Ordinary levels;GCSE, General Certificate of Secondary Education; CSE, Certificate of Secondary Education, A levels, Advanced levels; AS levels, Advanced Subsidiary levels; NVQ, National Vocational Qualification; HND, Higher National Diploma; HNC, Higher National Certificate; **P*-value <0.05

**Table S10.** General characteristics of participants included in and excluded from the current analysis

|  | **Participants, No. ()** | | ***p*-Value** |
| --- | --- | --- | --- |
|  | **Participants with complete covariates data (N=367,912)** | **Excluded participants due to missing data (N=134,475)** |  |
| Demographic characteristics |  |  |  |
| Age |  |  |  |
| Median (IQR), years | 57.0 (13.0) | 59.0 (13.0) | <0.001 |
| Missing | 0 (0.0) | 1 (0.0) |  |
| Sex |  |  |  |
| Male | 174758 (47.5) | 54318 (40.4) | <0.001 |
| Female | 193154 (52.5) | 80157 (59.6) |  |
| Ethnic |  |  |  |
| White | 347340 (94.4) | 125250 (93.1) | 0.495 |
| Non-White | 20572 (5.6) | 7347 (5.5) |  |
| Missing | 0 (0.0) | 1878 (1.4) |  |
| Body mass index |  |  |  |
| <25 kg/m^2^ | 122319 (33.2) | 42669 (31.7) | <0.001 |
| ≥25, <30 kg/m^2^ | 156326 (42.5) | 55745 (41.5) |  |
| ≥30 kg/m^2^ | 87503 (23.8) | 34720 (25.8) |  |
| Missing | 1764 (0.5) | 1341 (1.0) |  |
| Socio-economic characteristics |  |  |  |
| Thompson deprivation index |  |  |  |
| Low Deprivation | 73400 (20.0) | 27237 (20.3) | <0.001 |
| Mildly Deprivation | 74825 (20.3) | 25245 (18.8) |  |
| Moderately Deprivation | 74313 (20.2) | 26048 (19.4) |  |
| Highly Deprivation | 74562 (20.3) | 25784 (19.2) |  |
| Severely Deprivation | 70812 (19.2) | 29537 (22.0) |  |
| Missing | 0 (0) | 624 (0.5) |  |
| Qualifications |  |  |  |
| None | 54395 (14.8) | 30863 (23.0) | <0.001 |
| O levels, GCSEs, or CSEs | 198684 (54.0) | 54825 (40.8) |  |
| A levels or AS levels | 16131 (4.4) | 5555 (4.1) |  |
| NVQ, HND, HNC, or other professional qualification | 44635 (12.1) | 15927 (11.8) |  |
| College or university degree | 54067 (14.7) | 17175 (12.8) |  |
| Missing | 0 (0) | 10130 (7.5) |  |
| Employment status |  |  |  |
| Employed | 224137 (60.9) | 65579 (48.8) | <0.001 |
| Retired | 115306 (31.3) | 51050 (38.0) |  |
| Unemployed, home maker, or other | 28469 (7.7) | 14896 (11.1) |  |
| Missing | 0 (0) | 2950 (2.2) |  |
| Household income |  |  |  |
| <£18 000 | 83942 (22.8) | 13235 (9.8) | 0.004 |
| £18 000–£30 999 | 93847 (25.5) | 14304 (10.6) |  |
| £31 000–£51 999 | 95569 (26.0) | 15179 (11.3) |  |
| ≥£52 000 | 94554 (25.7) | 14617 (10.9) |  |
| Missing | 0 (0) | 77140 (57.4) |  |
| Lifestyle and medical history |  |  |  |
| The MET minutes per week for all activities |  |  |  |
| Median (IQR), minutes | 1770 (2741) | 1770 (2808) | 0.324 |
| Missing | 59567 (16.2) | 40536 (30.1) |  |
| Diabetes history |  |  |  |
| No | 349443 (95.0) | 126730 (94.2) | <0.001 |
| Yes | 18469 (5.0) | 7745 (5.8) |  |
| Hypertension history |  |  |  |
| No | 271657 (73.8) | 96491 (71.8) | <0.001 |
| Yes | 96255 (26.2) | 37984 (28.2) |  |
| Current somking |  |  |  |
| No | 329648 (89.6) | 118457 (88.1) | <0.001 |
| Yes | 38255 (10.4) | 15135 (11.3) |  |
| Missing | 9 (0.0) | 883 (0.7) |  |
| Current drinking |  |  |  |
| No | 27056 (7.4) | 14173 (10.5) | <0.001 |
| Yes | 340845 (92.6) | 119415 (88.8) |  |
| Missing | 11 (0.0) | 887 (0.7) |  |
| Environmental factors |  |  |  |
| Greenspace percentage within 300m buffer |  |  |  |
| Median (IQR), % | 29.7 (31.5) | 28.7 (29.5) | <0.001 |
| Greenspace percentage within 1000m buffer |  |  |  |
| Median (IQR), % | 41.8 (32.9) | 40.1 (31.0) | <0.001 |
| PM_2.5_ |  |  |  |
| Median (IQR), μg/m^3^ | 9.93 (1.3) | 9.97 (1.3) | <0.001 |
| Missing | 4250 (1.2) | 37046 (27.5) |  |
| PM_10_ |  |  |  |
| Median (IQR), μg/m^3^ | 16.0 (1.8) | 16.1 (1.8) | <0.001 |
| Missing | 4250 (1.2) | 37046 (27.5) |  |
| NO_2_ |  |  |  |
| Median (IQR), μg/m^3^ | 26.1 (9.9) | 26.6 (9.3) | <0.001 |
| Missing | 4036 (1.1) | 3340 (2.5) |  |

Note: IQR, interquartile range; Qualifications: O levels, Ordinary levels;GCSE, General Certificate of Secondary Education; CSE, Certificate of Secondary Education, A levels, Advanced levels; AS levels, Advanced Subsidiary levels; NVQ, National Vocational Qualification; HND, Higher National Diploma; HNC, Higher National Certificate;

MET minutes, metabolic equivalent of task minutes are obtained by multiplying the MET value of physical activity by its duration; PM_2.5_, particles with an aerodynamic diameter of ≤ 2.5 μm; PM_10_, particles with an aerodynamic diameter of ≤ 10 μm; NO_2_, nitrogen dioxide.

**Table S11.** The association between the percentage of greenspace within 300-m and 1000-m buffers and the incidence of ocular and adnexal diseases (including more covariates)

| **OAD subtypes** | | **GS% within 300m buffer** | | **GS% within 1000m buffer** | |
| --- | --- | --- | --- | --- | --- |
|  |  | HRb (95% CI) | ***P*-value** | HR (95% CI) | ***P*-value** |
| Disorders of lens (n=22683) | Q1a | Ref. | | | |
|  | Q2 | 0.979 (0.944, 1.017) | 0.276 | 0.987 (0.950, 1.025) | 0.492 |
|  | Q3 | 0.996 (0.960, 1.034) | 0.849 | 1.003 (0.965, 1.042) | 0.881 |
|  | Q4 | 0.933 (0.897, 0.970) | <0.001 | 0.920 (0.884, 0.958) | <0.001 |
| Disorders of choroid and retina (n=8130) | Q1 | Ref. | | | |
|  | Q2 | 0.992 (0.932, 1.055) | 0.797 | 0.987 (0.927, 1.051) | 0.693 |
|  | Q3 | 1.017 (0.956, 1.082) | 0.595 | 0.989 (0.927, 1.054) | 0.728 |
|  | Q4 | 0.961 (0.901, 1.026) | 0.234 | 0.944 (0.883, 1.009) | 0.089 |
| Disorders of eyelid, lacrimal system and orbit (n=9247) | Q1 | Ref. | | | |
|  | Q2 | 1.029 (0.969, 1.093) | 0.350 | 1.100 (1.035, 1.169) | 0.002 |
|  | Q3 | 1.155 (1.089, 1.225) | <0.001 | 1.215 (1.142, 1.292) | <0.001 |
|  | Q4 | 1.080 (1.016, 1.149) | 0.014 | 1.142 (1.071, 1.217) | <0.001 |
| Disorders of conjunctiva (n=3911) | Q1 | Ref. | | | |
|  | Q2 | 0.999 (0.910, 1.097) | 0.988 | 1.201 (1.092, 1.320) | <0.001 |
|  | Q3 | 1.122 (1.020, 1.235) | 0.018 | 1.117 (1.008, 1.237) | 0.035 |
|  | Q4 | 1.041 (0.914, 1.186) | 0.549 | 1.183 (1.040, 1.345) | 0.010 |
| Disorders of ocular muscles, binocular movement, accommodation and refraction (n=4741) | Q1 | Ref. | | | |
|  | Q2 | 0.972 (0.895, 1.056) | 0.499 | 0.954 (0.878, 1.037) | 0.266 |
|  | Q3 | 1.041 (0.959, 1.130) | 0.341 | 0.936 (0.851, 1.030) | 0.178 |
|  | Q4 | 0.964 (0.885, 1.051) | 0.408 | 0.924 (0.755, 1.132) | 0.446 |
| Glaucoma (n=5152) | Q1 | Ref. | | | |
|  | Q2 | 1.007 (0.930, 1.091) | 0.856 | 1.032 (0.951, 1.119) | 0.450 |
|  | Q3 | 1.032 (0.943, 1.130) | 0.489 | 1.038 (0.944, 1.141) | 0.440 |
|  | Q4 | 0.886 (0.715, 1.099) | 0.271 | 0.936 (0.752, 1.165) | 0.555 |
| Disorders of vitreous body and globe (n=3380) | Q1 | Ref. | | | |
|  | Q2 | 0.871 (0.788, 0.962) | 0.007 | 0.953 (0.862, 1.053) | 0.343 |
|  | Q3 | 1.026 (0.926, 1.137) | 0.625 | 0.941 (0.845, 1.049) | 0.272 |
|  | Q4 | 1.056 (0.904, 1.233) | 0.494 | 0.970 (0.839, 1.122) | 0.680 |
| Disorders of sclera, cornea, iris and ciliary body (n=2631) | Q1 | Ref. | | | |
|  | Q2 | 1.012 (0.901, 1.137) | 0.842 | 1.140 (1.015, 1.280) | 0.027 |
|  | Q3 | 1.078 (0.960, 1.210) | 0.204 | 1.051 (0.932, 1.187) | 0.416 |
|  | Q4 | 1.015 (0.899, 1.145) | 0.813 | 0.974 (0.859, 1.105) | 0.684 |
| Visual disturbances and blindness (n=4485) | Q1 | Ref. | | | |
|  | Q2 | 1.011 (0.930, 1.100) | 0.790 | 1.003 (0.923, 1.091) | 0.940 |
|  | Q3 | 1.045 (0.961, 1.137) | 0.301 | 0.981 (0.899, 1.071) | 0.668 |
|  | Q4 | 1.000 (0.915, 1.093) | 0.993 | 1.008 (0.920, 1.103) | 0.872 |

Note: The longitudinal study population consists of 240,684 individuals.

OAD, ocular and adnexal diseases; GS%, percentage of greenspace; HR, hazard ratio; CI, confidence interval; Ref, reference.

^a^The ranges for Q1-Q4 for GS% in the 300m buffer were “0-18.4, 18.4-31.3, 31.3-51.0, 51.0-99.2,” and for the 1000m buffer, they were “0-29.0, 29.0-44.0, 44.0-62.6, 62.6-99.2,” respectively.

^b^The models were adjusted for age, gender, ethnicity, indeices of thompson deprivation, employment status, household income, qualifications, BMI, alcohol status, somking status, diabetes history, and hypertension history.

**Table S12.** The association between the percentage of greenspace within 300-m and 1000-m buffers and the incidence of ocular and adnexal diseases (after excluding incidents within two-years follow-up)

| **OAD subtypes** | | **GS% within 300m buffer** | | **GS% within 1000m buffer** | |
| --- | --- | --- | --- | --- | --- |
|  |  | HRb (95% CI) | ***P*-value** | HR (95% CI) | ***P*-value** |
| Disorders of lens (n=20591) | Q1a | Ref. | | | |
|  | Q2 | 0.997 (0.958, 1.036) | 0.862 | 0.981 (0.942, 1.021) | 0.346 |
|  | Q3 | 0.999 (0.961, 1.039) | 0.956 | 0.983 (0.935, 1.034) | 0.515 |
|  | Q4 | 0.939 (0.901, 0.978) | 0.002 | 0.840 (0.732, 0.965) | 0.014 |
| Disorders of choroid and retina (n=7092) | Q1 | Ref. | | | |
|  | Q2 | 1.009 (0.942, 1.082) | 0.789 | 0.988 (0.922, 1.059) | 0.740 |
|  | Q3 | 1.031 (0.962, 1.104) | 0.392 | 0.981 (0.914, 1.054) | 0.601 |
|  | Q4 | 0.931 (0.866, 1.001) | 0.054 | 0.902 (0.838, 0.972) | 0.007 |
| Disorders of eyelid, lacrimal system and orbit (n=7127) | Q1 | Ref. | | | |
|  | Q2 | 1.011 (0.944, 1.082) | 0.761 | 1.092 (1.019, 1.170) | 0.013 |
|  | Q3 | 1.143 (1.070 1.222) | <0.001 | 1.210 (1.128, 1.297) | <0.001 |
|  | Q4 | 1.058 (0.987, 1.134) | 0.115 | 1.140 (1.060, 1.225) | <0.001 |
| Disorders of conjunctiva (n=2722) | Q1 | Ref. | | | |
|  | Q2 | 1.001 (0.897, 1.118) | 0.985 | 1.142 (1.021, 1.278) | 0.021 |
|  | Q3 | 1.070 (0.960, 1.194) | 0.223 | 1.062 (0.939, 1.202) | 0.337 |
|  | Q4 | 1.056 (0.944, 1.183) | 0.341 | 1.167 (0.995, 1.369) | 0.058 |
| Disorders of ocular muscles, binocular movement, accommodation and refraction (n=4336) | Q1 | Ref. | | | |
|  | Q2 | 0.972 (0.892, 1.059) | 0.519 | 0.931 (0.854, 1.015) | 0.106 |
|  | Q3 | 1.036 (0.952, 1.128) | 0.412 | 0.940 (0.850, 1.039) | 0.226 |
|  | Q4 | 0.956 (0.874, 1.045) | 0.322 | 0.855 (0.679, 1.077) | 0.183 |
| Glaucoma (n=4401) | Q1 | Ref. | | | |
|  | Q2 | 1.037 (0.953, 1.129) | 0.399 | 1.037 (0.951, 1.132) | 0.411 |
|  | Q3 | 1.046 (0.961, 1.139) | 0.302 | 1.047 (0.951, 1.153) | 0.346 |
|  | Q4 | 0.990 (0.906, 1.081) | 0.816 | 1.064 (0.928, 1.221) | 0.374 |
| Disorders of vitreous body and globe (n=2696) | Q1 | Ref. | | | |
|  | Q2 | 0.822 (0.736, 0.918) | 0.001 | 0.941 (0.842, 1.051) | 0.278 |
|  | Q3 | 0.967 (0.862, 1.084) | 0.563 | 0.908 (0.804, 1.027) | 0.124 |
|  | Q4 | 1.023 (0.855, 1.224) | 0.802 | 0.975 (0.820, 1.160) | 0.775 |
| Disorders of sclera, cornea, iris and ciliary body (n=2310) | Q1 | Ref. | | | |
|  | Q2 | 0.974 (0.865, 1.096) | 0.659 | 1.100 (0.978, 1.237) | 0.112 |
|  | Q3 | 1.041 (0.926, 1.169) | 0.505 | 1.018 (0.901, 1.151) | 0.775 |
|  | Q4 | 0.982 (0.869, 1.109) | 0.767 | 0.944 (0.831, 1.073) | 0.381 |
| Visual disturbances and blindness (n=3799) | Q1 | Ref. | | | |
|  | Q2 | 1.006 (0.918, 1.104) | 0.892 | 1.002 (0.916, 1.097) | 0.957 |
|  | Q3 | 0.996 (0.886, 1.119) | 0.940 | 0.993 (0.904, 1.091) | 0.891 |
|  | Q4 | 0.817 (0.581, 1.151) | 0.248 | 0.991 (0.899, 1.093) | 0.862 |

Note: The longitudinal study population consists of 234,083 individuals.

OAD, ocular and adnexal diseases; GS%, percentage of greenspace; HR, hazard ratio; CI, confidence interval; Ref, reference.

^a^The ranges for Q1-Q4 for GS% in the 300m buffer were “0-18.4, 18.4-31.3, 31.3-51.0, 51.0-99.2,” and for the 1000m buffer, they were “0-28.9, 28.9-44.0, 44.0-62.6, 62.6-99.2,” respectively.

^b^The models were adjusted for age, gender, ethnicity, indeices of thompson deprivation, employment status, household income and qualifications.

**Table S13.** The association between the percentage of greenspace within 300-m and 1000-m buffers and the incidence of ocular and adnexal diseases (after excluding multi-OAD patients)

| **OAD subtypes** | | **GS% within 300m buffer** | | **GS% within 1000m buffer** | |
| --- | --- | --- | --- | --- | --- |
|  |  | HRb (95% CI) | ***P*-value** | HR (95% CI) | ***P*-value** |
| Disorders of lens (n=10427) | Q1a | Ref. | | | |
|  | Q2 | 0.996 (0.942, 1.052) | 0.877 | 1.004 (0.948, 1.063) | 0.892 |
|  | Q3 | 0.975 (0.923, 1.031) | 0.373 | 1.036 (0.974, 1.102) | 0.258 |
|  | Q4 | 0.933 (0.881, 0.988) | 0.017 | 0.954 (0.874, 1.042) | 0.292 |
| Disorders of choroid and retina (n=2310) | Q1 | Ref. | | | |
|  | Q2 | 1.093 (0.972, 1.229) | 0.136 | 1.046 (0.929, 1.177) | 0.456 |
|  | Q3 | 1.106 (0.983, 1.244) | 0.093 | 1.074 (0.951, 1.212) | 0.250 |
|  | Q4 | 1.020 (0.902, 1.153) | 0.757 | 0.994 (0.876, 1.128) | 0.928 |
| Disorders of eyelid, lacrimal system and orbit (n=5479) | Q1 | Ref. | | | |
|  | Q2 | 1.057 (0.978, 1.143) | 0.163 | 1.131 (1.044, 1.225) | <0.001 |
|  | Q3 | 1.192 (1.104, 1.287) | <0.001 | 1.307 (1.207, 1.416) | <0.001 |
|  | Q4 | 1.137 (1.050, 1.231) | 0.002 | 1.204 (1.108, 1.308) | <0.001 |
| Disorders of conjunctiva (n=2373) | Q1 | Ref. | | | |
|  | Q2 | 1.009 (0.897, 1.135) | 0.886 | 1.202 (1.067, 1.353) | 0.002 |
|  | Q3 | 1.097 (0.976, 1.232) | 0.120 | 1.141 (1.008, 1.291) | 0.037 |
|  | Q4 | 1.142 (1.015, 1.286) | 0.028 | 1.257 (1.109, 1.423) | <0.001 |
| Disorders of ocular muscles, binocular movement, accommodation and refraction (n=335) | Q1 | Ref. | | | |
|  | Q2 | 1.157 (0.848, 1.580) | 0.358 | 0.831 (0.615, 1.122) | 0.226 |
|  | Q3 | 1.415 (1.046, 1.915) | 0.024 | 0.920 (0.678, 1.248) | 0.593 |
|  | Q4 | 1.024 (0.731, 1.435) | 0.891 | 0.760 (0.546, 1.057) | 0.103 |
| Glaucoma (n=2151) | Q1 | Ref. | | | |
|  | Q2 | 1.023 (0.904, 1.157) | 0.721 | 1.068 (0.926, 1.232) | 0.364 |
|  | Q3 | 1.089 (0.964, 1.230) | 0.173 | 1.235 (0.963, 1.584) | 0.096 |
|  | Q4 | 1.061 (0.936, 1.202) | 0.355 | 1.698 (0.656, 4.396) | 0.275 |
| Disorders of vitreous body and globe (n=1123) | Q1 | Ref. | | | |
|  | Q2 | 0.827 (0.691, 0.991) | 0.039 | 1.104 (0.920, 1.325) | 0.286 |
|  | Q3 | 1.028 (0.867, 1.220) | 0.750 | 1.124 (0.925, 1.367) | 0.239 |
|  | Q4 | 1.163 (0.981, 1.380) | 0.082 | 1.350 (1.054, 1.728) | 0.017 |
| Disorders of sclera, cornea, iris and ciliary body (n=397) | Q1 | Ref. | | | |
|  | Q2 | 1.078 (0.811, 1.433) | 0.604 | 1.307 (0.982, 1.739) | 0.067 |
|  | Q3 | 1.286 (0.975, 1.696) | 0.075 | 1.292 (0.959, 1.740) | 0.093 |
|  | Q4 | 1.019 (0.753, 1.378) | 0.904 | 1.221 (0.895, 1.666) | 0.208 |
| Visual disturbances and blindness (n=2051) | Q1 | Ref. | | | |
|  | Q2 | 1.069 (0.944, 1.211) | 0.295 | 1.068 (0.942, 1.211) | 0.303 |
|  | Q3 | 1.130 (0.997, 1.281) | 0.055 | 1.132 (0.994, 1.289) | 0.061 |
|  | Q4 | 1.119 (0.981, 1.276) | 0.093 | 1.165 (1.018, 1.333) | 0.026 |

Note: The longitudinal study population consists of 225,968 individuals.

OAD, ocular and adnexal diseases; GS%, percentage of greenspace; HR, hazard ratio; CI, confidence interval; Ref, reference.

^a^The ranges for Q1-Q4 for GS% in the 300-m buffer were “0-18.4, 18.4-31.3, 31.3-51.0, 51.0-99.2,” and for the 1000-m buffer, they were “0-28.9, 28.9-44.0, 44.0-62.6, 62.6-99.2,” respectively.

^b^The models were adjusted for age, gender, ethnicity, indeices of thompson deprivation, employment status, household income and qualifications.

**Table S14.** The association between the percentage of greenspace within 300-m and 1000-m buffers and the incidence of ocular and adnexal diseases (competing risks model).

| **OAD subtypes** | | **GS% within 300m buffer** | | **GS% within 1000m buffer** | |
| --- | --- | --- | --- | --- | --- |
|  |  | Fine-Gray model  (aHR^b^ [95% CI]) | Cause-specific hazards model (aHR [95% CI]) | Fine-Gray model  aHR (95% CI) | Cause-specific hazards model (aHR [95% CI]) |
| Disorders of lens (n=22787) | Q1a | Ref. | | | |
|  | Q2 | 0.981 (0.946, 1.020) | 0.985 (0.949, 1.022) | 0.990 (0.954, 1.030) | 0.990 (0.954, 1.028) |
|  | Q3 | 0.999 (0.962, 1.040) | 0.999 (0.963, 1.037) | 1.000 (0.965, 1.040) | 1.004 (0.966, 1.043) |
|  | Q4 | 0.928 (0.893, 0.965)* | 0.931 (0.896, 0.968)* | 0.917 (0.881, 0.954)* | 0.917 (0.881, 0.954)* |
| Disorders of choroid and retina (n=8160) | Q1 | Ref. | | | |
|  | Q2 | 1.004 (0.944, 1.068) | 0.997 (0.935, 1.063) | 0.995 (0.935, 1.059) | 0.988 (0.926, 1.054) |
|  | Q3 | 1.030 (0.968, 1.096) | 1.010 (0.947, 1.077) | 1.001 (0.939, 1.068) | 0.985 (0.922, 1.053) |
|  | Q4 | 0.964 (0.904, 1.028) | 0.935 (0.874, 1.000)* | 0.945 (0.884, 1.011) | 0.921 (0.859, 0.987)* |
| Disorders of eyelid, lacrimal system and orbit (n=9293) | Q1 | Ref. | | | |
|  | Q2 | 1.038 (0.978, 1.101) | 1.039 (0.979, 1.103) | 1.104 (1.039, 1.172)* | 1.105 (1.040, 1.174)* |
|  | Q3 | 1.164 (1.098, 1.233)* | 1.164 (1.098, 1.234)* | 1.224 (1.152, 1.301)* | 1.226 (1.153, 1.303)* |
|  | Q4 | 1.084 (1.020, 1.153)* | 1.082 (1.018, 1.150)* | 1.141 (1.071, 1.217)* | 1.139 (1.069, 1.214)* |
| Disorders of conjunctiva (n=3936) | Q1 | Ref. | | | |
|  | Q2 | 1.006 (0.918, 1.102) | 1.005 (0.917, 1.102) | 1.207 (1.101, 1.323)* | 1.208 (1.100, 1.326)* |
|  | Q3 | 1.129 (1.032, 1.235)* | 1.129 (1.027, 1.241)* | 1.128 (1.025, 1.242)* | 1.127 (1.018, 1.247)* |
|  | Q4 | 1.081 (0.984, 1.186) | 1.079 (0.943, 1.234) | 1.214 (1.101, 1.338)* | 1.198 (1.053, 1.363)* |
| Disorders of ocular muscles, binocular movement, accommodation and refraction (n=4757) | Q1 | Ref. | | | |
|  | Q2 | 0.965 (0.890, 1.047) | 0.963 (0.887, 1.044) | 0.937 (0.864, 1.015) | 0.936 (0.862, 1.016) |
|  | Q3 | 1.025 (0.946, 1.111) | 1.028 (0.948, 1.114) | 0.924 (0.850, 1.003) | 0.918 (0.834, 1.010) |
|  | Q4 | 0.951 (0.875, 1.035) | 0.955 (0.877, 1.039) | 0.855 (0.784, 0.932)* | 0.827 (0.666, 1.028) |
| Glaucoma (n=5165) | Q1 | Ref. | | | |
|  | Q2 | 1.015 (0.938, 1.098) | 1.010 (0.932, 1.093) | 1.031 (0.952, 1.116) | 1.028 (0.948, 1.114) |
|  | Q3 | 1.063 (0.984, 1.150) | 1.032 (0.943, 1.129) | 1.053 (0.971, 1.143) | 1.031 (0.939, 1.133) |
|  | Q4 | 1.007 (0.928, 1.092) | 0.885 (0.715, 1.096) | 1.038 (0.955, 1.129) | 0.934 (0.751, 1.161) |
| Disorders of vitreous body and globe (n=3397) | Q1 | Ref. | | | |
|  | Q2 | 0.863 (0.783, 0.952)* | 0.870 (0.788, 0.961)* | 0.955 (0.866, 1.053) | 0.955 (0.865, 1.055) |
|  | Q3 | 0.999 (0.909, 1.098) | 1.018 (0.919, 1.127) | 0.943 (0.853, 1.042) | 0.943 (0.847, 1.051) |
|  | Q4 | 0.966 (0.876, 1.065) | 1.029 (0.882, 1.200) | 0.978 (0.884, 1.082) | 0.982 (0.848, 1.138) |
| Disorders of sclera, cornea, iris and ciliary body (n=2643) | Q1 | Ref. | | | |
|  | Q2 | 1.018 (0.912, 1.137) | 1.018 (0.910, 1.139) | 1.117 (1.001, 1.247)* | 1.128 (1.009, 1.262)* |
|  | Q3 | 1.077 (0.966, 1.202) | 1.080 (0.966, 1.208) | 1.051 (0.938, 1.178) | 1.046 (0.931, 1.175) |
|  | Q4 | 1.009 (0.900, 1.132) | 1.015 (0.904, 1.141) | 0.974 (0.865, 1.098) | 0.981 (0.869, 1.109) |
| Visual disturbances and blindness (n=4512) | Q1 | Ref. | | | |
|  | Q2 | 1.018 (0.938, 1.105) | 1.018 (0.937, 1.106) | 0.994 (0.915, 1.079) | 1.000 (0.920, 1.086) |
|  | Q3 | 1.043 (0.960, 1.134) | 1.045 (0.961, 1.136) | 0.974 (0.894, 1.062) | 0.980 (0.899, 1.069) |
|  | Q4 | 0.989 (0,906, 1.080) | 0.988 (0.905, 1.080) | 0.989 (0.904, 1.082) | 0.989 (0.904, 1.082) |

Note: OAD, ocular and adnexal diseases; GS%, percentage of greenspace; HR, hazards ratio; CI, confidence interval; Ref, reference.

^a^The ranges for Q1-Q4 for GS% in the 300-m buffer were “0-18.4, 18.4-31.3, 31.3-51.0, 51.0-99.2,” and for the 1000-m buffer, they were “0-29.0, 29.0-44.0, 44.0-62.6, 62.6-99.2,” respectively. ^b^The models were adjusted for age, gender, ethnicity, indeices of thompson deprivation, employment status, household income, and qualifications.

**P*-value <0.05

**Table S15.** The association between the percentage of greenspace within 300-m and 1000-m buffers and the incidence of ocular and adnexal diseases after Multiple Imputation by Chained Equations (MICE)

| **OAD subtypes** | | **GS% within 300m buffer** | | **GS% within 1000m buffer** | |
| --- | --- | --- | --- | --- | --- |
|  |  | aHRa (95% CI) | ***P*-value** | aHR (95% CI) | ***P*-value** |
| Disorders of lens (n=33064) | Q1 | Ref. | | | |
|  | Q2 | 0.961 (0.927, 0.997) | 0.033 | 0.976 (0.941, 1.012) | 0.189 |
|  | Q3 | 0.976 (0.944, 1.009) | 0.151 | 0.964 (0.919, 1.012) | 0.126 |
|  | Q4 | 0.884 (0.849, 0.921) | <0.001 | 0.867 (0.834, 0.900) | <0.001 |
| Disorders of choroid and retina (n=11425) | Q1 | Ref. | | | |
|  | Q2 | 1.000 (0.945, 1.060) | 0.988 | 0.996 (0.943, 1.052) | 0.886 |
|  | Q3 | 0.995 (0.928, 1.067) | 0.880 | 0.993 (0.931, 1.059) | 0.829 |
|  | Q4 | 0.908 (0.851, 0.970) | 0.004 | 0.905 (0.849, 0.963) | 0.002 |
| Disorders of eyelid, lacrimal system and orbit (n=13517) | Q1 | Ref. | | | |
|  | Q2 | 1.046 (0.995, 1.100) | 0.078 | 1.164 (1.104, 1.228) | <0.001 |
|  | Q3 | 1.202 (1.140, 1.267) | <0.001 | 1.243 (1.171, 1.320) | <0.001 |
|  | Q4 | 1.095 (1.034, 1.159) | 0.002 | 1.182 (1.111, 1.257) | <0.001 |
| Disorders of conjunctiva (n=6459) | Q1 | Ref. | | | |
|  | Q2 | 0.856 (0.766, 0.957) | 0.009 | 1.220 (1.060, 1.403) | 0.010 |
|  | Q3 | 1.023 (0.899, 1.164) | 0.711 | 1.131 (0.984, 1.301) | 0.079 |
|  | Q4 | 1.098 (0.923, 1.308) | 0.262 | 1.367 (1.135, 1.646) | 0.004 |
| Disorders of ocular muscles, binocular movement, accommodation and refraction (n=6323) | Q1 | Ref. | | | |
|  | Q2 | 0.973 (0.906, 1.044) | 0.440 | 0.960 (0.893, 1.033) | 0.273 |
|  | Q3 | 1.026 (0.948, 1.111) | 0.526 | 0.928 (0.853, 1.010) | 0.085 |
|  | Q4 | 0.926 (0.856, 1.002) | 0.057 | 0.781 (0.644, 0.948) | 0.012 |
| Glaucoma (n=7323) | Q1 | Ref. | | | |
|  | Q2 | 1.021 (0.917, 1.137) | 0.689 | 1.051 (0.973, 1.136) | 0.201 |
|  | Q3 | 1.042 (0.940, 1.156) | 0.418 | 1.012 (0.914, 1.121) | 0.812 |
|  | Q4 | 0.940 (0.722, 1.224) | 0.633 | 0.948 (0.742, 1.213) | 0.663 |
| Disorders of vitreous body and globe (n=4656) | Q1 | Ref. | | | |
|  | Q2 | 0.906 (0.809, 1.015) | 0.086 | 0.954 (0.873, 1.043) | 0.300 |
|  | Q3 | 1.016 (0.911, 1.134) | 0.768 | 0.987 (0.896, 1.087) | 0.786 |
|  | Q4 | 1.012 (0.858, 1.193) | 0.889 | 1.001 (0.874, 1.145) | 0.992 |
| Disorders of sclera, cornea, iris and ciliary body (n=3777) | Q1 | Ref. | | | |
|  | Q2 | 1.045 (0.936, 1.166) | 0.427 | 1.104 (1.001, 1.217) | 0.048 |
|  | Q3 | 1.035 (0.896, 1.196) | 0.619 | 1.075 (0.969, 1.193) | 0.171 |
|  | Q4 | 0.956 (0.853, 1.073) | 0.440 | 0.982 (0.862, 1.120) | 0.784 |
| Visual disturbances and blindness (n=6274) | Q1 | Ref. | | | |
|  | Q2 | 0.982 (0.907, 1.064) | 0.658 | 0.981 (0.898, 1.071) | 0.660 |
|  | Q3 | 0.994 (0.913, 1.083) | 0.889 | 0.957 (0.876, 1.045) | 0.321 |
|  | Q4 | 0.906 (0.826, 0.995) | 0.039 | 0.930 (0.852, 1.016) | 0.105 |

Note: OAD, ocular and adnexal diseases; GS%, percentage of greenspace; HR, hazard ratio; CI, confidence interval; Ref, reference; Q1-Q4, the first quartile to the fourth quartile. After excluding baseline patients using questionnaire (502,387→397,063) and electronic health record (397,063→338,282) criteria, the final analytic sample comprised 338,282 participants.

Multiple imputation by chained equations were conducted by the “MICE” package; number of multiple imputations: 5; maximum number of iterations for each imputation: 5; The estimates were pooled by the Rubin rule.

^a^The models were adjusted for age, gender, ethnicity, indices of thompson deprivation, employment status, household income, and qualifications.

**Table S16.** The association between the percentage of greenspace within 300-m and 1000-m buffers and the incidence of ocular and adnexal diseases after controlling for the central effect (random term)

| **OAD subtypes** | | **GS% within 300m buffer** | | **GS% within 1000m buffer** | |
| --- | --- | --- | --- | --- | --- |
|  |  | aHRb (95% CI) | ***P*-value** | aHR (95% CI) | ***P*-value** |
| Disorders of lens (n=22787) | Q1a | Ref. | | | |
|  | Q2 | 0.974 (0.938, 1.011) | 0.171 | 0.974 (0.937, 1.013) | 0.193 |
|  | Q3 | 0.982 (0.945, 1.021) | 0.356 | 0.984 (0.944, 1.025) | 0.429 |
|  | Q4 | 0.924 (0.888, 0.962) | <0.001 | 0.910 (0.871, 0.949) | <0.001 |
| Disorders of choroid and retina (n=8160) | Q1 | Ref. | | | |
|  | Q2 | 0.989 (0.926, 1.055) | 0.732 | 0.980 (0.917, 1.047) | 0.544 |
|  | Q3 | 0.995 (0.932, 1.063) | 0.889 | 0.973 (0.908, 1.043) | 0.436 |
|  | Q4 | 0.927 (0.865, 0.994) | 0.032 | 0.913 (0.849, 0.982) | 0.014 |
| Disorders of eyelid, lacrimal system and orbit (n=9293) | Q1 | Ref. | | | |
|  | Q2 | 0.996 (0.937, 1.058) | 0.896 | 1.018 (0.956, 1.083) | 0.586 |
|  | Q3 | 1.070 (1.007, 1.136) | 0.029 | 1.096 (1.028, 1.169) | 0.005 |
|  | Q4 | 1.012 (0.950, 1.078) | 0.711 | 1.041 (0.973, 1.114) | 0.241 |
| Disorders of conjunctiva (n=3936) | Q1 | Ref. | | | |
|  | Q2 | 1.000 (0.902, 1.109) | 0.990 | 1.134 (1.020, 1.261) | 0.021 |
|  | Q3 | 1.113 (0.980, 1.264) | 0.099 | 1.076 (0.939, 1.234) | 0.290 |
|  | Q4 | 1.132 (0.965, 1.329) | 0.130 | 1.272 (1.074, 1.506) | 0.005 |
| Disorders of ocular muscles, binocular movement, accommodation and refraction (n=4757) | Q1 | Ref. | | | |
|  | Q2 | 0.979 (0.901, 1.065) | 0.624 | 0.856 (0.768, 0.955) | 0.005 |
|  | Q3 | 1.056 (0.971, 1.149) | 0.204 | 0.774 (0.656, 0.913) | 0.002 |
|  | Q4 | 1.021 (0.934, 1.116) | 0.649 | 0.665 (0.528, 0.839) | <0.001 |
| Glaucoma (n=5165) | Q1 | Ref. | | | |
|  | Q2 | 1.112 (1.011, 1.224) | 0.029 | 1.137 (1.030, 1.255) | 0.011 |
|  | Q3 | 1.254 (1.102, 1.428) | <0.001 | 1.262 (1.103, 1.443) | <0.001 |
|  | Q4 | 1.285 (1.084, 1.525) | 0.003 | 1.355 (1.138, 1.613) | <0.001 |
| Disorders of vitreous body and globe (n=3397) | Q1 | Ref. | | | |
|  | Q2 | 0.964 (0.859, 1.081) | 0.530 | 1.112 (0.987, 1.251) | 0.080 |
|  | Q3 | 1.208 (1.043, 1.399) | 0.012 | 1.246 (1.065, 1.457) | 0.006 |
|  | Q4 | 1.264 (1.049, 1.523) | 0.014 | 1.458 (1.198, 1.773) | <0.001 |
| Disorders of sclera, cornea, iris and ciliary body (n=2643) | Q1 | Ref. | | | |
|  | Q2 | 1.016 (0.906, 1.139) | 0.786 | 1.116 (0.994, 1.254) | 0.063 |
|  | Q3 | 1.071 (0.955, 1.202) | 0.239 | 1.025 (0.906, 1.159) | 0.698 |
|  | Q4 | 1.011 (0.896, 1.141) | 0.860 | 0.969 (0.852, 1.103) | 0.636 |
| Visual disturbances and blindness (n=4512) | Q1 | Ref. | | | |
|  | Q2 | 1.028 (0.945, 1.118) | 0.525 | 0.986 (0.904, 1.074) | 0.740 |
|  | Q3 | 1.052 (0.965, 1.146) | 0.252 | 0.965 (0.881, 1.058) | 0.448 |
|  | Q4 | 0.980 (0.894, 1.074) | 0.662 | 0.953 (0.866, 1.049) | 0.330 |

Note: OAD, ocular and adnexal diseases; GS%, percentage of greenspace; HR, hazard ratio; CI, confidence interval; Ref, reference. Effect estimates were calculated by the “coxme” package and “frailtypack” package in R software.

^a^The ranges for Q1-Q4 for GS% in the 300-m buffer were “0-18.4, 18.4-31.3, 31.3-51.0, 51.0-99.2,” and for the 1000-m buffer, they were “0-29.0, 29.0-44.0, 44.0-62.6, 62.6-99.2,” respectively.

^b^The models were adjusted for age, gender, ethnicity, indices of thompson deprivation, employment status, household income, and qualifications.

**Table S17.** The population attributable fraction of the percentage of greenspace within 300-m and 1000-m buffers for ocular and adnexal diseases with significant association to greenspace

| **OAD subtypes** | | **GS% within 300m buffer** | | **GS% within 1000m buffer** | |
| --- | --- | --- | --- | --- | --- |
|  |  | PAFb, % (95% CI) | ***P*-value** | PAF, % (95% CI) | ***P*-value** |
| Disorders of lens (n=22787) | Q4a | Ref. | | | |
|  | Q1 | 1.72 (0.85, 2.76) | <0.001 | 2.08 (1.19, 3.32) | <0.001 |
|  | Q2 | 1.37 (0.53, 2.41) | 0.004 | 1.85 (0.96, 2.94) | <0.001 |
|  | Q3 | 1.70 (0.88, 2.78) | <0.001 | 2.17 (1.32, 3.24) | <0.001 |
| Disorders of choroid and retina (n=8160) | Q4 | Ref. | | | |
|  | Q1 | 1.63 (0.03, 3.57) | 0.048 | 1.98 (0.47, 3.91) | 0.020 |
|  | Q2 | 1.56 (0.02, 3.31) | 0.050 | 1.70 (0.16, 3.46) | 0.038 |
|  | Q3 | 1.87 (0.25, 3.61) | 0.022 | 1.64 (0.07, 3.32) | 0.046 |
| Disorders of eyelid, lacrimal system and orbit (n=9293) | Q1 | Ref. | | | |
|  | Q2 | 0.95 (-0.44, 2.46) | 0.180 | 2.38 (0.94, 4.24) | 0.006 |
|  | Q3 | 3.52 (2.48, 5.54) | <0.001 | 4.60 (3.65, 7.11) | <0.001 |
|  | Q4 | 1.90 (0.56, 3.65) | 0.024 | 3.06 (1.73, 5.03) | <0.001 |
| Disorders of conjunctiva (n=3936) | Q1 | Ref. | | | |
|  | Q2 | 0.13 (-2.31, 2.46) | 0.900 | 4.31 (2.41, 7.58) | <0.001 |
|  | Q3 | 2.85 (0.75, 5.62) | 0.014 | 2.81 (0.41, 5.93) | 0.020 |
|  | Q4 | 1.82 (-1.48, 5.47) | 0.306 | 4.13 (1.46, 8.16) | 0.006 |

Note: OAD, ocular and adnexal diseases; GS%, percentage of greenspace; PAF, population attributable fraction; CI, confidence interval; Ref, reference.

The confidence interval was calculated by Bootstrap Method (N=1000).

^a^The ranges for Q1-Q4 for GS% in the 300m buffer were “0-18.4, 18.4-31.3, 31.3-51.0, 51.0-99.2,” and for the 1000m buffer, they were “0-29.0, 29.0-44.0, 44.0-62.6, 62.6-99.2,” respectively.

^b^PAF = [(*HR* - 1) / *HR*] * *Pe*; *Pe* represents the proportion of the population exposed to the risk factor.

**Code S1.** The code for the main data analysis in this study (based on R)

**Part 1**.The association between the percentage of greenspace within 300m and 1000m buffer and the incidence of ocular and adnexal diseases

*#PH_cox model*

*library(survival)*

*#H00-H06*

*cox_mode3<-coxph(Surv(H00_H06_followtime_year,stat.H00.H06)~Greenspace_300m_Q+strata(Age)+strata(Sex)+ethnic+TDI_Q+Household_income_Q+Qualifications_Q+strata(Employment_status_Q),data=data_cleaned)*

*#Note:The analysis is similar for other disease types, for instance：*

*cox_mode3<-coxph(Surv(H10_H13_followtime_year,stat.H10.H13)~Greenspace_300m_Q+tt(Greenspace_300m_Q)+strata(Age)+strata(Sex)+ethnic+strata(TDI_Q)+ Household_income_Q+strata(Qualifications_Q)+Employment_status_Q,data=data_cleaned)*

**Part 2**.The population attributable fraction

*#PAF*

*P_e_Q2 <- mean(data_cleaned$Greenspace_300m_Q == "2")*

*P_e_Q3 <- mean(data_cleaned$Greenspace_300m_Q == "3")*

*P_e_Q4 <- mean(data_cleaned$Greenspace_300m_Q == "4")*

*#Enter the corresponding HR value*

*PAF_Q2 <- ((HR_Q2 - 1) / HR_Q2) * P_e_Q2*

*PAF_Q3 <- ((HR_Q3 - 1) / HR_Q3) * P_e_Q3*

*PAF_Q4 <- ((HR_Q4 - 1) / HR_Q4) * P_e_Q4*

*#Note:The analysis is similar for the percentage of greenspace within 1000m buffer*

**Part 3**.Mediated effect decomposition of four-way decomposition method.

*install.packages("devtools")*

*library("devtools")*

*devtools::install_github("BS1125/CMAverse")*

*library("CMAverse")*

*datamet <- data_cleaned[!is.na(data_cleaned$MET),]*

*set.seed(123)*

*met <- cmest(data = datamet, model = "rb", outcome = "H25_H28_followtime_year",event = "stat.H25.H28",*

*exposure = "Greenspace_300m_Q", mediator = "MET", basec = c("Age", "Sex","ethnic","TDI_Q","Employment_status_Q","Household_income_Q", "Qualifications_Q", “UK.Biobank.assessment.centre...Instance.0”),*

*EMint = T, mreg = list("linear"), yreg = "coxph",*

*astar = 0, a = 4,*

*mval = list(0),*

*estimation = "imputation",*

*inference = "bootstrap")*

**Part 4**.Sensitivity analyzes.

#Competing risks models

*library(prodlim)*

*library(riskRegression)*

*library(survival)*

*library(dplyr)*

*library(lava)*

*#Cause-specific hazards model*

*csc_model <- CSC(*

*Hist(H00_H06_followtime_year, event.H00.H06) ~*

*Greenspace_300m_Q +strata(Age) +strata(Sex) + strata(Employment_status_Q) +ethnic +TDI_Q +*

*Household_income_Q + Qualifications_Q,*

*data = data_cleaned,*

*fitter = "coxph",*

*cause = 1 )*

*#Fine-Gary model*

*fgr_full_H15_H22_1000 <- FGR(*

*Hist(H15_H22_followtime_year, event.H15.H22) ~*

*Greenspace_1000m_Q+strata(Age)+Sex +ethnic+ strata(TDI_Q)+ strata(Household_income_Q)+*

*strata(Qualifications_Q)+strata(Employment_status_Q),*

*data = data_cleaned,*

*cause = 1)*

*#MICE*

*library(mice)*

*library(dplyr)*

*library(VIM)*

*library(survival)*

*data_mice <- data_mice %>%*

*mutate(*

*Sex = factor(Sex),*

*ethnic = factor(ethnic),*

*Employment_status_Q = factor(Employment_status_Q),*

*Qualifications_Q = factor(Qualifications_Q),*

*Household_income_Q = ordered(Household_income_Q),*

*Age = as.numeric(Age),*

*TDI_Q = factor(TDI_Q),*

*Greenspace_300m_Q = factor(Greenspace_300m_Q),*

*Greenspace_1000m_Q = factor(Greenspace_1000m_Q))*

*ini <- mice(data_mice, maxit = 0, print = T)*

*meth <- ini$meth*

*set.seed(202312)*

*imp <- mice(data_mice,*

*m = 5,*

*maxit = 5,*

*method = meth,*

*seed = 202312,*

*printFlag = TRUE)*

*fit_model3 <- with(imp,*

*coxph(Surv(H00_H06_followtime_year, stat.H00.H06) ~*

*Greenspace_300m_Q +*

*strata(Age) +*

*strata(Sex) +*

*ethnic +*

*TDI_Q +*

*Household_income_Q +*

*Qualifications_Q +*

*strata(Employment_status_Q)))*

**Supplementary Methods.** Four-way decomposition method

The excess relative risk (ERR) was used to evaluate the total effect of green space on specific OAD subtypes, which could be decomposed into four components [1] (Eq. 1).


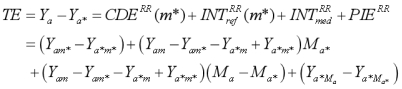


(Eq.1)

Where a and a* denoted the exposure and reference level of the percentage of greenspace (GS%); m* denoted the reference level of mediators, which was set to 0 in this study.
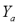
 and
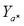
 were potential outcomes when
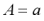
 and
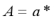
;
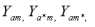
and
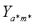
 were potential outcomes for different levels of GS% (a, a*) and mediators (m, m*). The ERR of each component can be calculated by the following formulas [2]:


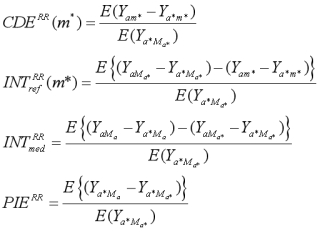


(Eq.2)

Then the proportion of the total effect attributed to each component can be expressed by the ratios EER(CDE)/[RR(TE)-1], EER(INTref)/[RR(TE)-1], EER(INTmed)/[RR(TE)-1], EER(PIE)/[RR(TE)-1].

**Reference**

1. Valeri L, VanderWeele TJ. Mediation analysis allowing for exposure–mediator interactions and causal interpretation: theoretical assumptions and implementation with SAS and SPSS macros. *Psychological methods* 2013;**18(2)**:137
2. Li Z, Wu W, Huang Y, et al. Urban residential greenness and cancer mortality: Evaluating the causal mediation role of air pollution in a large cohort. Environ Pollut. 2024;**360**:124704. doi:10.1016/j.envpol.2024.124704.
